# An alternative recommendation for design eccentricity by consideration of uncoupled frequency ratio

**DOI:** 10.1038/s41598-024-75465-3

**Published:** 2024-12-28

**Authors:** Osman Akyürek, Hakan Ulutaş

**Affiliations:** 1 Department of Civil Engineering, Nevsehir Haci Bektasi Veli University, Nevsehir, Turkey; 2 Department of Civil Engineering, Mehmet Akif Ersoy University, Burdur, Turkey

**Keywords:** Torsional irregularity, Amplification factor, Accidental torsion, Uncoupled frequency ratio, Effective radius of gyration, Building code, ASCE 7–22, Natural hazards, Civil engineering

## Abstract

Civil structures are prone to dynamic loadings such as strong winds or ground excitations where torsion becomes an ongoing issue. This arises from a lack of coincidence of the center of mass (CM) and rigidity (CR), known as eccentricity. Seismic design codes often introduce two types of eccentricity: inherent (geometric) and accidental. To account for structural or ground motion uncertainties, an assumption-based solution is provided by many code provisions, which considers the accidental eccentricity as a percentage (5% or 10%) of the building length perpendicular to the direction of exposed ground motion. In this study, as an alternative way to the code design parameters, a new design eccentricity formula that considers the frequency ratio (torsional frequency/translation frequency) and an effective radius of gyration to account for torsional irregularity is considered. For the extended validation of the proposed method, eighteen model buildings with six different floor plans were chosen, representing low, medium-height, and high-rise buildings. Each floor plan had model buildings with three, seven, and twelve stories. The buildings were subjected to selected bidirectional earthquake ground motions and had time history analyses performed. The results of the proposed method were compared to code provision methods, obtained using equivalent lateral force procedures, and also to those obtained utilizing the time history analysis results. It was shown that the proposed method was more effective in estimating the impact of torsional eccentricity and provided a better understanding of its impact on structural dynamic characteristics.

**Supplementary Information:**

The online version contains supplementary material available at 10.1038/s41598-024-75465-3.

## Introduction

In structural design with developing technologies and methods, civil structures became taller and taller (slender) and more flexible day by day in the 21st century by using lighter materials and employing sophisticated modern control systems. This inclination leads the buildings to become more vulnerable to strong earthquakes and severe winds, especially for those architecturally and structurally having complex shapes that have torsion as a vital matter^1^.

During an earthquake loading, the structure has dynamic effects that induce inertia forces acting laterally through the CM whereas the load-resisting members withstand them from the CR; besides, those reversing forces are not coincident in many real-life structures. The non-coincidence CM and CR can produce eccentricities in two orthogonal directions that lead to an unwanted torsional response. This torsional sensitivity may lead to an increase in torsional coupling effects (aerodynamic loads), which can also increase eccentricity between CM and CR, especially once the torsional mode is dominant. The contribution of torsional coupling effects to lateral response can, for instance, cause an increase in up to 50% as compared to the relating symmetrical structure^2–4^.

The seismic response of the buildings is significantly affected by torsional irregularity, and it may lead to partial damage or total collapse of structures, which was observed in a few former earthquakes. For example, the 1985 Mexico earthquake is one of the most intensively investigated earthquakes. According to the earthquake field investigation by Scholl in 1989^5^, 177 buildings were damaged completely, and 85 buildings experienced a limited failure, 15% of which underwent the lateral-torsional coupling effect. For those 42%, torsionally coupled structures were corner buildings^6,7^.

Torsional irregularity has been a subject of extensive research, and new recommendations and recoveries are constantly being offered. The findings reveal that for a single-story building having two-way eccentricity under a unidirectional loading case, the torsional coupling effect can reduce the base shear, overturning moment, and roof displacement while increasing the base torque. Additionally, increasing eccentricity in the way perpendicular to the exposed earthquake excitation increases the torsional moment, while an increase in eccentricity in the way of earthquake excitation lessens the torsional moment. Furthermore, the coupled frequency ratio (fundamental torsional frequency/translation frequency ($\:{\Omega}_{x}$ or $\:{\Omega}_{y}$) is a crucial factor in determining the torsional coupling effect, particularly as it gets values between 0.75 and 1.25^8,9^.

It has been found that when studying the effects of twisting forces on multi-story buildings, it is more practical to use a one-story building to calculate the torsional effects^10–12^. In cases where bi-directional earthquake excitations are present, the coupling effects tend to increase as compared to unidirectional earthquake excitations. Using unidirectional excitations may not be sufficient for accurately estimating the torsional response, as the parameters that control the torsional response, such as stiffness, radius of gyration, and location of the center of stiffness, can be significantly altered under bi-directional excitations^13–18^. While many experimental studies are still required to examine and verify the effects of torsional coupling, there are a few studies available that have already been conducted^19,20^.

To guarantee the safety and stability of structures, seismic design codes mandate that torsional effects must be taken into account, even in cases where no inherent eccentricity exists. To achieve this, design eccentricities - both inherent and accidental - are taken into account for each direction. Inherent eccentricity is calculated as the absolute distance between the CM and CR in the structural plan, whereas accidental eccentricity can be defined as the absolute distance between inherent and calculated-design eccentricities. Design parameters are also provided in seismic design codes to account for accidental eccentricity and ensure that buildings can withstand torsional effects. However, it is important to note that this consideration may not be sufficient to address all design parameters, as accidental eccentricity can be unpredictable and subject to change due to structural and ground motion uncertainty. To learn more about studies conducted on ground motion uncertainty, interested readers can refer to sources such as^21–25^.

In conclusion, even if there is no geometric eccentricity due to the design, significant lateral-torsion effects can be seen in the structures due to uncertainties in the structural design and earthquake ground motion. The eccentricity that causes this situation is called accidental eccentricity. Many building codes provide assumption-based solution methods. Here, the accidental eccentricity effect is taken into account by multiplying the torsional irregularity coefficient by a percentage (5% or 10%) of the length of the structure perpendicular to the earthquake direction. The current code provision, ASCE 7–22, might be insufficient to represent the effects of torsion^26,27^. Therefore, an alternative design eccentricity formula was proposed^28^, which includes the frequency ratio (torsional frequency/translation frequency for each direction) and an effective radius of rotation to reconsider the torsional irregularity. In this study, the extended performance evaluation of the proposed design eccentricity was made. To this purpose; six different floor plans which have three-story representing low structures, seven-story representing medium-height structures, and twelve-story representing high-rise structures each, and a total of eighteen buildings’ dynamic analyses (THAs) are performed in case of selected bidirectional historical earthquake ground motions taken place in Turkey. The obtained results are compared to ASCE 7–22 and TBEC-2018. According to the results, the torsional irregularity in structures can be predicted more conveniently in the existing structures and structures to be built. In addition, it can provide a more realistic solution to explain how torsional irregularity change structural dynamic characteristic.

## Torsional provisions in ASCE 7–22 AND TBEC-2018 code provision

**Fig. 1:**
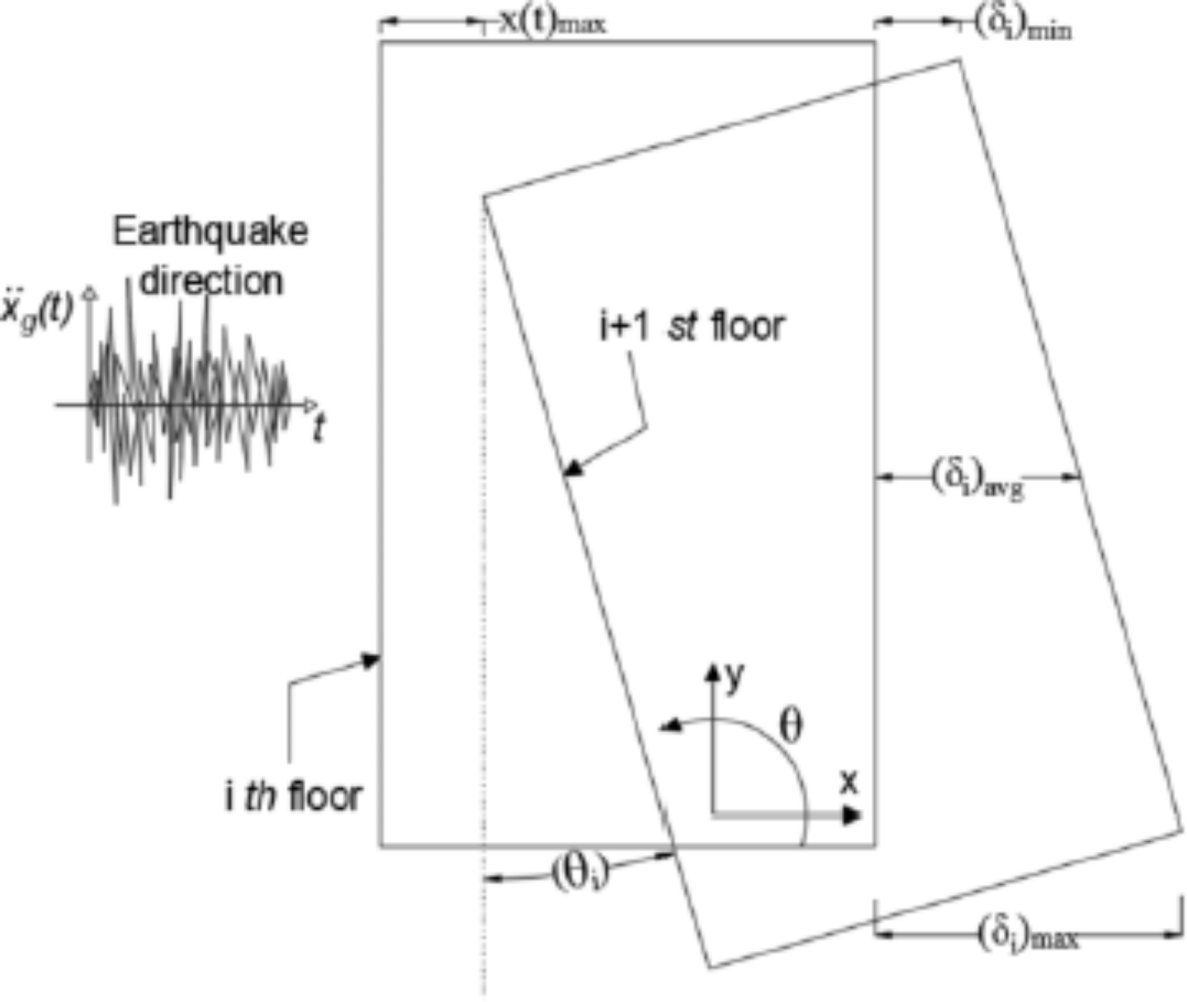
Representation of torsional irregularity by illustrating maximum and average displacement at the maximum (x(t)_max_) time history response at the i-th floor.

There are two methods of analyzing accidental eccentricity: dynamic analysis and static analysis. According to ASCE 7–22 (American Society of Civil Engineers Standard 7 in Sect. 12.8^29^ and TEBC-2018 (Turkish Building Earthquake Code in 2018) Sect. 4.7^30^, the static analysis procedure is recommended. This procedure is preferred because it is simpler and more practical than the time history analysis procedure. The Equivalent Lateral Force (ELF) procedure is utilized to calculate equivalent shear forces at each floor and total shear force at the base level. It does not require computing the center of rigidity (CR). This analysis makes use of an assumption-based solution that takes into consideration accidental eccentricity by changing the CM at each floor from its actual location by a distance equal to 5% of the building plan length which is perpendicular to the earthquake ground motion direction (ASCE 7–22 Sect. 12.8.4.2 and TBEC-2018 Sect. 4.5.10). The obtained torsional moment, -resulting from the shift in CM, is applied at the CM. In both ASCE 7–22 and TEBC-2018, the definition of torsional irregularity and design eccentricity are the same, except for using different torsional irregularity coefficient notations like $\:{{A}_{x}=\left(\frac{{\eta\:}_{bi}}{1.2}\right)}^{2}$where $\:{\eta\:}_{bi}$ is the rate of the maximum to average displacement in the related floor. This study employs the notations used in the ASCE 7–22 design code. The torsional irregularity definition can be categorized into three cases as follows:

1. If the value of $\:{A}_{x}$ is less than 1, then no torsional irregularity exists and, in that case, $\:{A}_{x}$ is equal to 1.
2. If the $\:{A}_{x}$ gets the values between 1 and 3, then torsional irregularity exists, see Fig. 1 and the torsional amplification factor is defined as such:

1 $$\:1.0\le\:{A}_{x}={\left(\frac{{\delta\:}_{max}}{1.2{\delta\:}_{avg}}\right)}^{2}\le\:3.0$$

In which, $\:{(\delta\:}_{max}$ and $\:{\delta\:}_{min})$ are respectively the maximum and minimum displacements, and $\:\left({\delta\:}_{avg}\right)$ is the average displacement at the building edge that gives the maximum change in response at level *i + 1* floor.

(3) If it is higher than 3, extreme torsional irregularity exists and, in that case, $\:{A}_{x}\:$is taken as 3.

The code-based design eccentricities, $\:{(e}_{d1})$ and $\:{(e}_{d2})$ for earthquake loading through in the x-direction are respectively calculated as such:

2 $$\:{e}_{d1}=1.0{e}_{n}+0.05{L}_{y}{A}_{x}$$

3 $$\:{e}_{d2}=1.0{e}_{n}-0.05{L}_{y}{A}_{x}$$

Here, $\:{e}_{n}$ is the inherent (geometric) eccentricity, and *L*_*x*_ and *L*_*y*_ represent the plan dimensions in the x- and y-directions respectively. It is also important to note that whereas the accidental eccentricity gets amplified by the torsional amplification factor (A_x_) and 5% of the building length in the plan (*L*_*y*_) perpendicular to the earthquake direction, the inherent eccentricity remains constant according to code provision, as shown in Eqs. (2)-(3).

4 $$\:{M}_{d1i}={e}_{d1i} \cdot {F}_{e1i}$$

5 $$\:{M}_{d2i}={e}_{d2i} \cdot {F}_{e2i}$$

The torsional moments affecting the design of a building’s structure on a given story, $\:\left({M}_{d1i}\right)$ and $\:{(M}_{d2i})$, are determined by the equivalent lateral forces at each level, multiplied by the moment derived from inherent $\:\:{(e}_{n})$, and accidental, $\:{(e}_{ac})$ eccentricities, see Eqs. (4)-(5). The accidental torsional moment is calculated by shifting the building’s mass to a distance equal to the torsional amplification factor, $\:{(A}_{x})$ multiplied by 5% of the plan dimension (*L*_*y*_). This method of shifting the center of mass to consider accidental torsion is broadly known for both static and dynamic analyses although it can alter the building’s dynamic characteristics. To avoid this, computing the torsional moment by ELF procedures can be a preferable and more robust method to account for the torsional effect without affecting the structural dynamic characteristics. This study employs the ELF procedure to validate the new recommendation of computing the design eccentricity.

## Structural dynamics & mathematical modeling

### Equation of motion

Two-way torsionally asymmetric one-story shear building, subjected to unidirectional or bidirectional earthquake ground motions in orthogonal directions, has three degrees of freedom for each floor, which are horizontal displacements at two orthogonal directions and a rotation around gravity at the center of the mass. The equation of motion for a single degree of freedom (SDOF) damped system, which is torsionally coupling, can be mathematically expressed in Eq. (6) and its state space representations as follows:

6 $$\:M\ddot{\delta\:}\left(t\right)+C\dot{\delta\:}\left(t\right)+K\delta\:\left(t\right)=-W{\ddot{x}}_{g}\left(t\right)$$

In which, mass matrix ($\:M)$, damping matrix $\:\left(C\right)$, stiffness matrix $\:\left(K\right)$, the modified input vector$\:(W$), earthquake input vector $\:\left({\ddot{x}}_{g}\right(t\left)\right)$, the displacement, velocity and acceleration vectors $\:(\delta\:\left(t\right),\:\dot{\delta\:}\left(t\right),\:\ddot{\delta\:}\left(t\right)$), radius of gyration ($\:r)$, and its components in the x direction $\:{(r}_{x})$ and y direction $\:{(r}_{y})$ are obtained as well as polar mass moment of inertia$\:\:\left({J}_{0}\right)$ and torsional stiffness$\:\:{(k}_{\theta\:})$ where $\:{(e}_{nx}\left)\:\text{a}\text{n}\text{d}\:{(e}_{nx}\right)$ are the x and y component of the inherent eccentricity as follows:

7 $$\:M=\left[\begin{array}{ccc}{m}_{x}&\:0&\:0\\\:0&\:{m}_{y}&\:0\\\:0&\:0&\:{J}_{0}\end{array}\right],\:K=\left[\begin{array}{ccc}{k}_{x}&\:0&\:-{e}_{ny}.{k}_{x}\\\:0&\:{k}_{y}&\:{e}_{nx}.{k}_{y}\\\:-{e}_{ny}.{k}_{x}&\:{e}_{nx}.{k}_{y}&\:{k}_{\theta\:}\end{array}\right]$$

8 $$\:W=\left[\begin{array}{c}{m}_{x}\\\:0\\\:0\end{array}\right],\:\delta\:\left(t\right)=\left[\begin{array}{c}{\delta\:}_{x}\left(t\right)\\\:{\delta\:}_{y}\left(t\right)\\\:\theta\:\left(t\right)\end{array}\right]$$

9 $$\:{r}_{x}=\frac{{L}_{x}}{\sqrt{12}},\:{r}_{y}=\frac{{L}_{y}}{\sqrt{12}},\:r=\sqrt{{{r}_{x}}^{2}+{{r}_{y}}^{2}}$$

10 $$\:{J}_{0}=m \cdot \:{r}^{2}$$

11 $$\:{k}_{\theta\:}={k}_{x} \cdot \frac{{{L}_{y}}^{2}}{4}+{k}_{y} \cdot \frac{{{L}_{x}}^{2}}{4}+{k}_{x}{e}_{ny}^{2}+{k}_{y}{e}_{nx\:}^{2}$$

### Effective radius of gyration

**Fig. 2:**
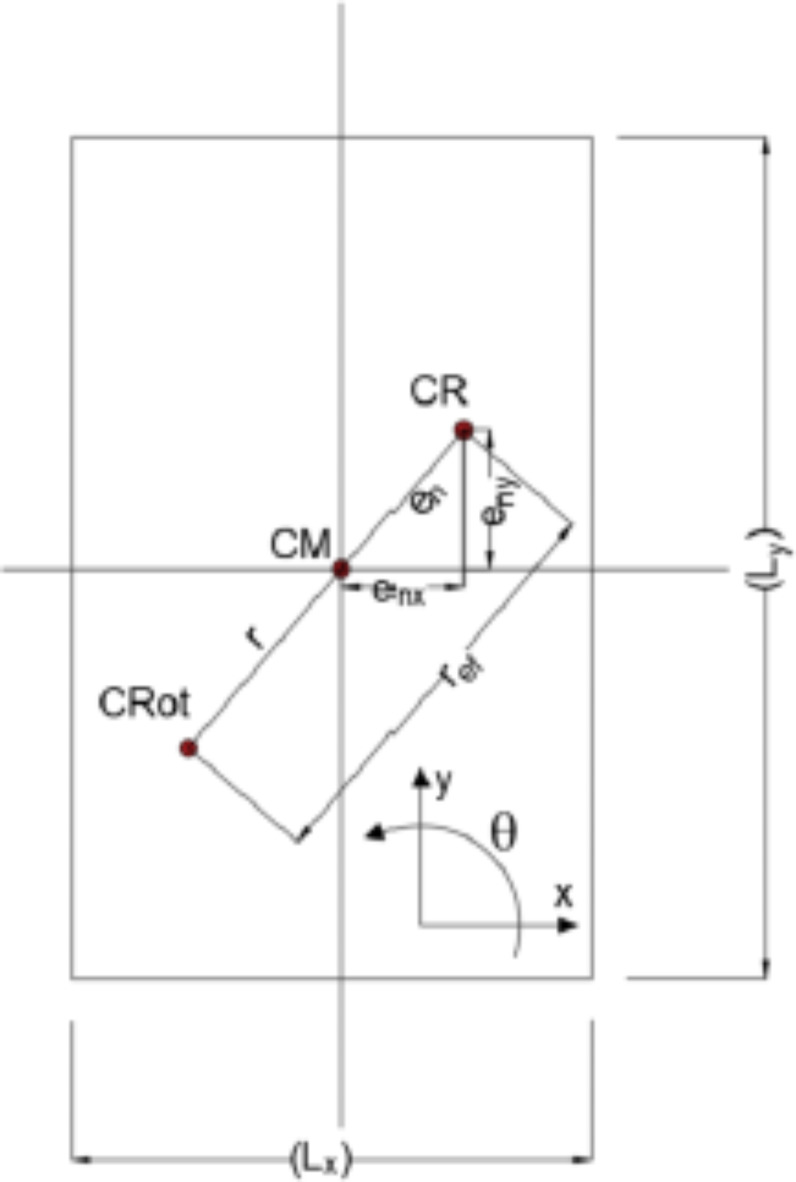
The effective radii of gyration in a floor plan.

The radius of gyration (r) can be calculated using Eq. 9 for a rectangular-shaped building. It can be simply defined as the absolute distance between the Center of Rotation (CRot) and the Center of Mass (CM) while the geometric eccentricity (e_n_) is the absolute distance between the CM and CR. This might not be an adequate definition, taking into account accidental torsional building responses in the case of Torsionally Irregular Buildings (TIBs). Because the TIBs give their reactions from the Center of Rigidity (CR), which does not coincide with the CM, once an earthquake loading strikes the building through the CM. The most critical situation happens when the radius of gyration is aligned with the geometric eccentricity, as seen in Fig. 2. For this reason, in order to stay on the safe side, the effective radius of gyration (r_ef_) is taken into account, instead of using only the radius of gyration, in calculating the design eccentricity. The mathematical expressions of the geometric eccentricity (e_n_) and the effective radius of gyration (r_ef_) are respectively given as follows:

12 $$\:{e}_{n}=\sqrt{{e}_{nx\:}^{2}+{e}_{ny\:}^{2}}$$

13 $$\:{\overrightarrow{r}}_{ef}=\overrightarrow{r}+{\overrightarrow{e}}_{n}$$

### Proposed design eccentricity

**Fig. 3:**
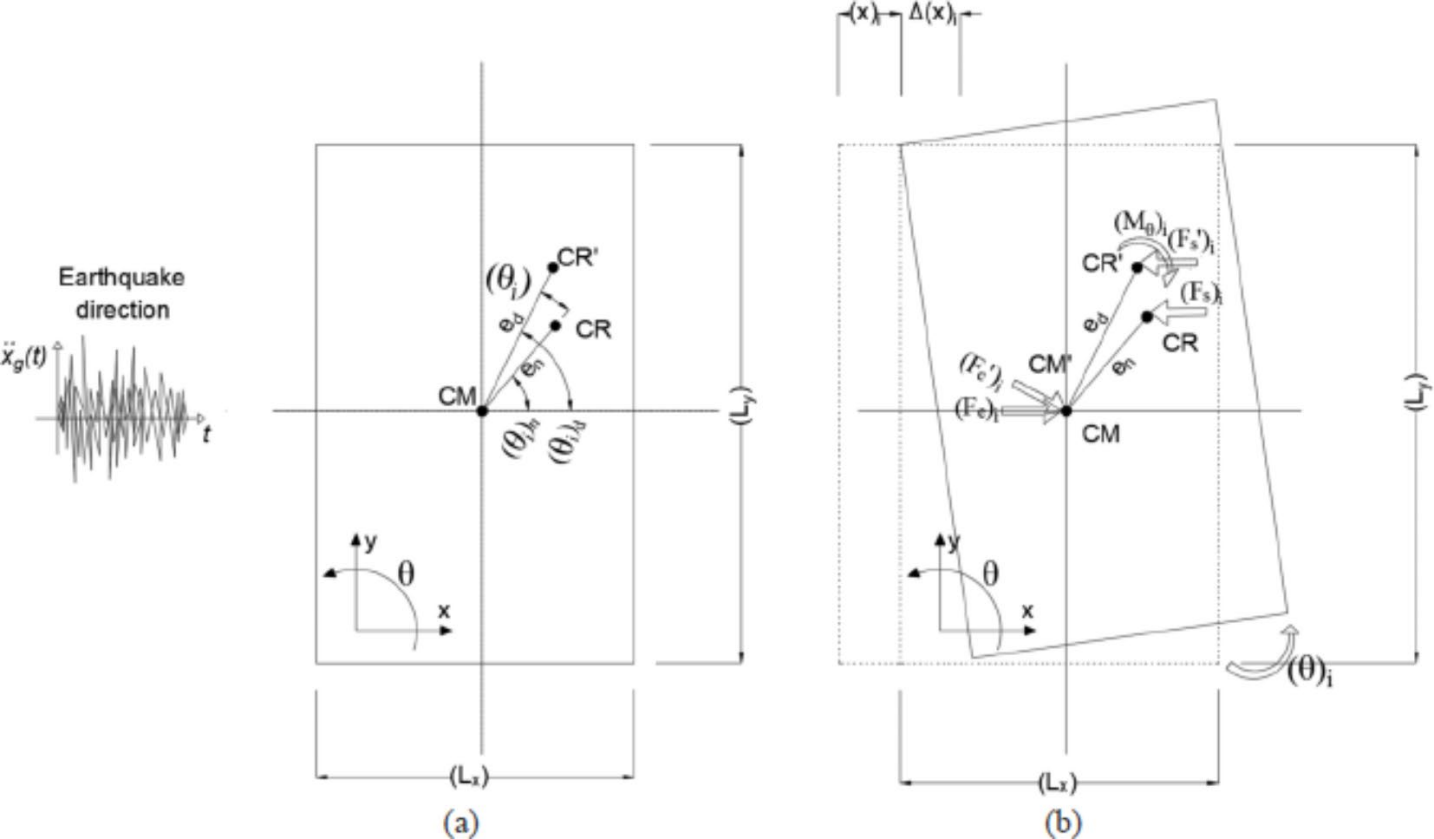
Illustration of the design and inherent eccentricities of a structure, including the accidental torsional response (**a**) and the equilibrium position before and after (‘) the applied force (**b**).

The definition and formula given for an alternative design eccentricity including frequency ratio, $\:,\:$here was proposed by the first author in his study^28^. For better understanding and clarification of the model, it is stated here again. Before the applied ground excitation, the structure is statically stable, see Fig. 3a. The structure gives the x-lateral and rotational responses after applied ground motion, as seen in Fig. 3b.

To obtain the eccentricity formula under the transitional ground excitation, the Newton second law is employed to compute the design eccentricity to the center of mass (CM).

14 $$\:\sum\:{F}_{i}=0$$

15 $$\:{F}_{e}={F}_{s}={k}_{x} \cdot x\left(t\right)$$

where $\:{(F}_{e}$) is the equivalent lateral earthquake shear force and $\:\left({F}_{s}\right)$ is the strain force in the direction where the earthquake excitation is applied.

16 $$\:\sum\:{M}_{i}=0$$

17 $$\:{F}_{s}{\prime\:} \cdot {e}_{dy}-{M}_{\theta\:}=0$$

where$\:\:{(F}_{s}{\prime\:})$ and $\:{(M}_{\theta\:})$ are, in order, the strain force and the torque (torsional moment) with respect to the CM including the effect of the accidental eccentricity.

18 $$\:{F}_{s}{\prime\:}={k}_{x} \cdot \left(x\left(t\right)+\varDelta\:x\left(t\right)\right)$$

19 $$\:{M}_{\theta\:}={k}_{\theta\:} \cdot \theta\:\left(t\right)=\left[\left({k}_{x} \cdot \frac{{{L}_{y}}^{2}}{4}+{k}_{y} \cdot \frac{{{L}_{x}}^{2}}{4}+{k}_{x}{e}_{ny}^{2}+{k}_{y}{e}_{nx\:}^{2}\right)\right] \cdot \theta\:\left(t\right)$$

Then, the design eccentricity ($\:{e}_{dy})$ is proportional between the ratio of torsional stiffness to the stiffness in the x-direction and torsional response to lateral response in the x-direction. It is given in Eq. 20.

20 $$\:{e}_{dy}=\frac{{k}_{\theta\:}}{{k}_{x}} \cdot \frac{\theta\:\left(t\right)}{\left(x\left(t\right)+{L}_{y}\theta\:\left(t\right)\right)}$$

The ratio between $\:{k}_{\theta\:}\:\text{a}\text{n}\text{d}{\:k}_{x}\:$can be written where the $\:{r}_{ef}$ is the effective radius of gyration as:

21 $$\:{\Omega}_{x}=\frac{{w}_{\theta\:}}{{w}_{x}}$$

22 $$\:{w}_{\theta\:}=\sqrt{\frac{{k}_{\theta\:}}{{{r}_{ef}}^{2} \cdot M}},\:{w}_{x}=\sqrt{\frac{{k}_{x}}{M}}$$

23 $$\:\frac{{k}_{\theta\:}}{{k}_{x}}={\left({\Omega}_{x} \cdot {r}_{ef}\right)}^{2}$$

Here $\:{w}_{x}$ and $\:{w}_{\theta\:}$ are respectively representing the first dominant mode (frequency in Hz) for the x-lateral direction and the $\:\theta\:$-torsional directions.

According to ASCE 7–22, torsional irregularity coefficient ($\:{A}_{x})$ is obtained by the square of the ratio of $\:{(\delta\:}_{max})$ over 1.2 times$\:{\:(\delta\:}_{avg})$. The maximum $\:{(\delta\:}_{max})$ and minimum $\:{(\delta\:}_{min})$ drift at the level of the floor, and torsional irregularity coefficient, ($\:{A}_{x})$, can be expressed as

24 $$\:{\delta\:}_{max}={x\left(t\right)}_{max}+{L}_{y}{\theta\:\left(t\right)}_{max}$$

25 $$\:{\delta\:}_{avg}={x\left(t\right)}_{max}+\frac{{L}_{y}}{2}{\theta\:\left(t\right)}_{max}$$

26 $$\:{\delta\:}_{avg}=\frac{({\delta\:}_{max}+{\delta\:}_{\text{m}\text{i}\text{n}})}{2}$$

27 $$\:{A}_{x}={\left(\frac{{\delta\:}_{max}}{1.2{\delta\:}_{avg}}\right)}^{2}$$

where $\:{x\left(t\right)}_{max}$ and $\:{\theta\:\left(t\right)}_{max}$ are the peak response of the time history analysis in the x- and $\:{\theta\:}$-direction. The ratio of the torsional response to the lateral response in the x-direction becomes

28 $$\:{x\left(t\right)}_{max}=\left(\frac{1-0.6\sqrt{{A}_{x}}}{1.2\sqrt{{A}_{x}}-1}\right){L}_{y}{\theta\:\left(t\right)}_{max}$$

When substituting Eq. 23 and Eq. 28 into Eq. 20, the design eccentricity under the ground motion in the x-direction can be determined by using Eq. 29.

29 $$\:{e}_{dy}=\frac{{k}_{\theta\:}}{{k}_{x}} \cdot \frac{\theta\:\left(t\right)}{\left(x\left(t\right)+{L}_{y}\theta\:\left(t\right)\right)}={\left({\Omega}_{x} \cdot {r}_{ef}\right)}^{2}\frac{\theta\:\left(t\right)}{\left[\left(\frac{1-0.6\sqrt{{A}_{x}}}{1.2\sqrt{{D}_{bi}}-1}\right)+1\right]{L}_{y}\theta\:\left(t\right)}\rightarrow{e}_{dy}=\frac{{\left({\Omega}_{x} \cdot {r}_{ef}\right)}^{2}}{{L}_{y}}\left(\frac{1.2\sqrt{{A}_{x}}-1}{0.6\sqrt{{A}_{x}}}\right)$$

For simplification, if $\:\varvec{\gamma\:}=\left(\frac{1.2\sqrt{{A}_{x}}-1}{0.6\sqrt{{A}_{x}}}\right)$ which is called Proposed Torsional Irregularity Coefficient (PTIC), then the design eccentricity equation becomes as

30 $$e_{{dy}} = \frac{{\left( {\Omega_{x} \cdot r_{{ef}} } \right)^{2} }}{{L_{y} }}\gamma$$

31 $$\:{e}_{dx}\cong\:{e}_{nx}$$

For the unidirectional earthquake loading case from the x-direction, the design eccentricity is defined as the sum of the geometric and accidental eccentricity in the y-direction. That is, the design eccentricity found in Eq. 30 is also equal to $\:{e}_{dy}={e}_{ny}+{e}_{acy}$. For the other direction, the design eccentricity in the x-direction is assumed to be equal to the geometric eccentricity, see Eq. 31. Similarly, when the structure is subjected to the earthquake excitation in the y-direction, the design eccentricity becomes as seen in Eq. 32.

32 $$e_{{dx}} = \frac{{\left( {\Omega_{y} \cdot r_{{ef}} } \right)^{2} }}{{L_{x} }}\gamma$$

33 $$\:{e}_{dy}\cong\:{e}_{ny}$$

It is defined as the sum of the geometric and accidental eccentricity in the y-direction. That is, the design eccentricity is also equal to $\:{e}_{dx}={e}_{nx}+{e}_{acx}$. For the other direction, it is assumed to be equal to the geometric eccentricity, see Eq. 33 where $\:{e}_{dx}$ and $\:{e}_{acx}$; and $\:{e}_{dy}$ and $\:{e}_{acy}$ are respectively the design and the accidental eccentricity of x-component and y-component of the design eccentricity.

**Fig. 4:**
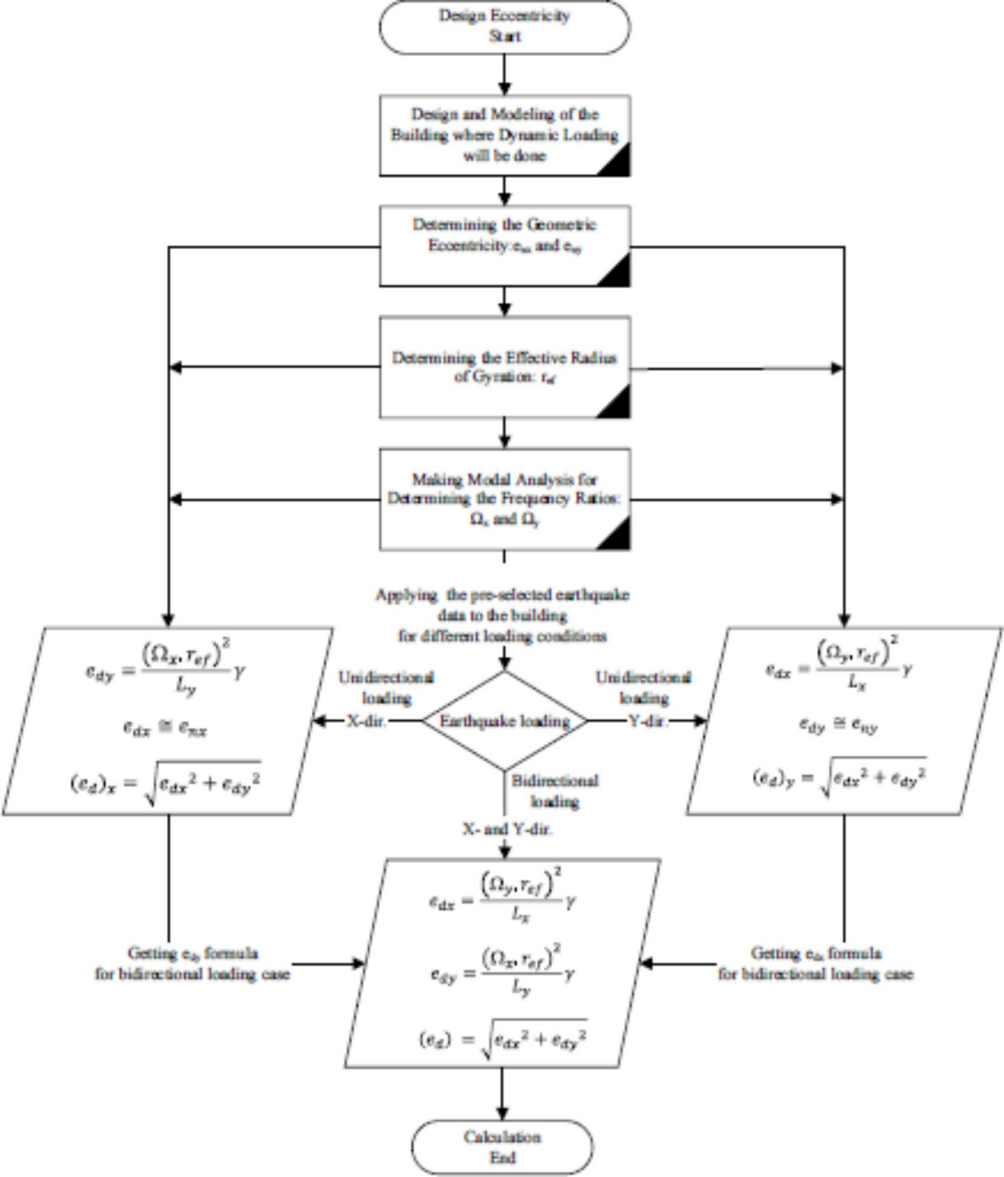
The design eccentricity for the building subjected to unidirectional and bi-directional ground motion.

Under bidirectional earthquake loading cases, the design eccentricities are found out by using the obtained design eccentricity Eq. 30 when the structure is subjected to the ground motion in the x-direction and Eq. 32 under the ground motion in the y-direction. Torsional irregularity coefficients,$\:\:{A}_{x}\:\text{o}\text{r}\:{A}_{y},$ are taken as calculated, i.e., when $\:{A}_{x}=0.9$, not taken 1 like in ASCE 7–22. The flaw scheme of the design eccentricities for uni- and bidirectional excitation are illustrated in Fig. 4.

### Torsional irregularity definition with PTIC (γ)

**Fig. 5:**
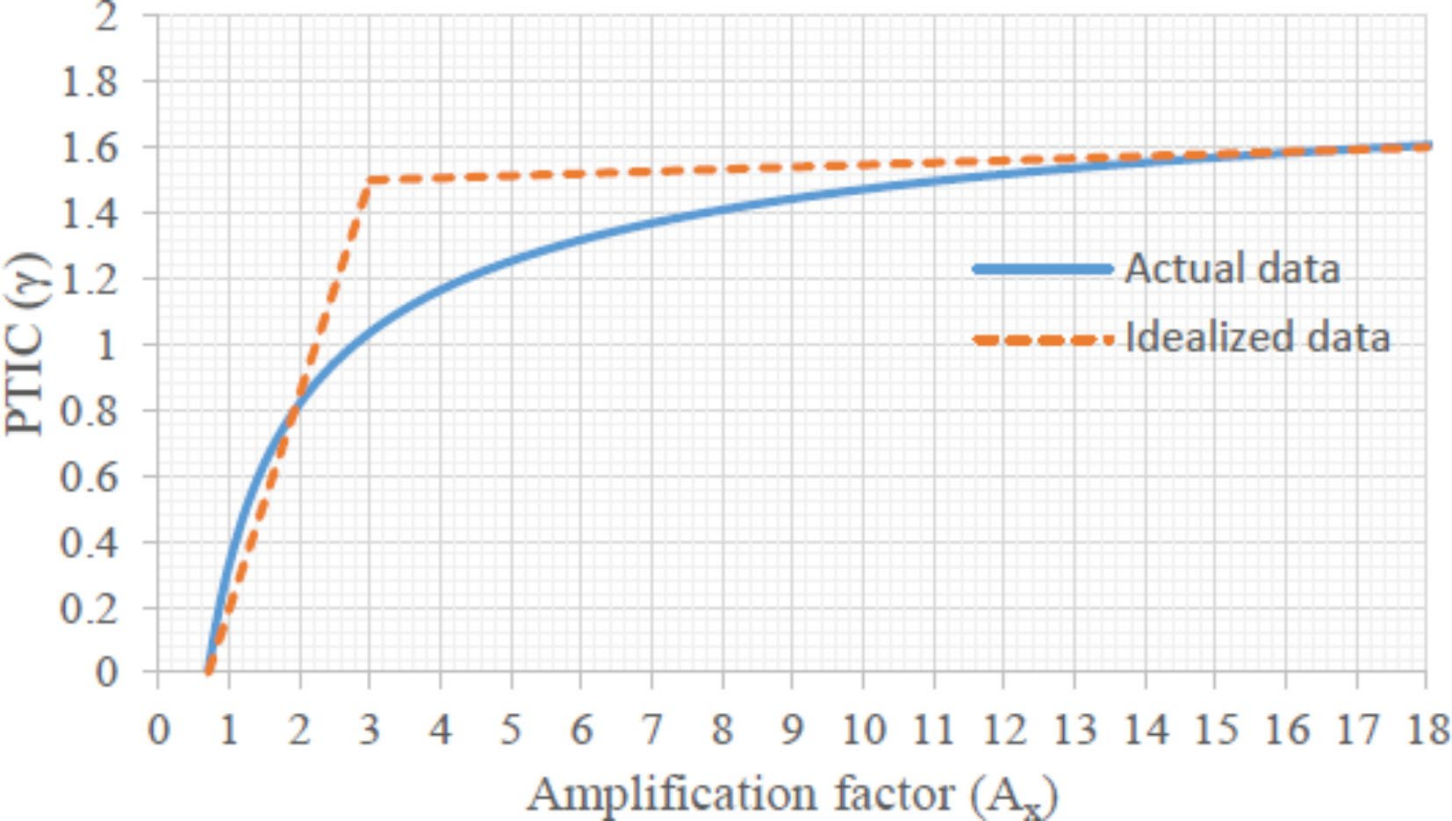
Torsional irregularity definition with γ coefficient.

With the definition of torsional irregularity in the ASCE 7–22, the PTIC (γ) can also be classified into three categories in a similar manner when it is idealized, see Fig. 5. If A_x_ is less than 0.7, there is no torsional irregularity. If A_x_ is between 1 and 3, torsional irregularity exists, and γ linearly increases up to 1.5. Finally, if A_x_ is greater than 3, there is extreme torsional irregularity, and γ remains around 1.5.

## Overview of model buildings

**Fig. 6:**
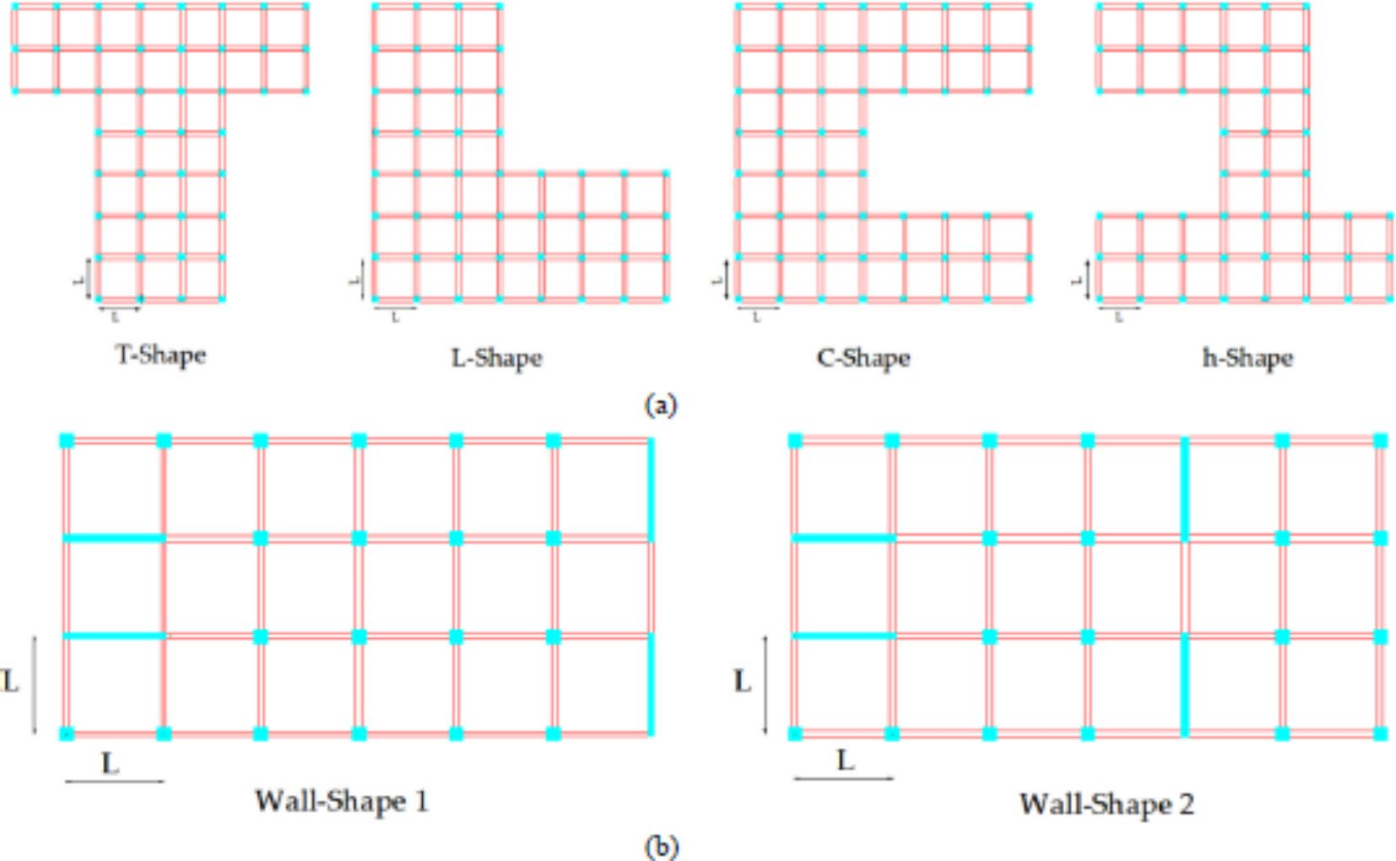
Floor plans of reinforced concrete frame model buildings; (**a**) only columns and beams are load-bearing, and (**b**) columns + beams and shear walls load carrying.

In this study for the verification of the proposed design eccentricity, a wide range of building layouts, which have both irregular geometry and regular geometry but experiencing eccentricity due to non-symmetrical shear wall placements within framed buildings, are selected, see Fig. 6a and b. Performance evaluations for six different floor plans that represent buildings with varying floor plans are made. A total of eighteen buildings are analyzed, including three-story buildings that represent low-rise structures, seven-story buildings that represent mid-rise structures, and twelve-story buildings that represent high-rise structures. The results of these analyses are compared to current regulations to determine the efficacy of our proposed method.

For each floor plan in all buildings, the span (L) is 5 m and all floor heights are 3 m. The dimensions of the column and beam sections are respectively 65 × 65 and 50 × 25 cm. The width of the shear wall along with the selected axis is 25 cm. The used concrete grade is C30. Dead, live, and infill wall load are respectively taken as 4.5, 2, and 4 kN/m^2^. The live load reduction coefficient is taken at 0.3. The constant damping ratio is employed and taken as 5%. Furthermore, all model buildings have been meticulously designed to meet the design criteria of the ASCE-7-22, ensuring the use of high-ductility load-bearing elements. Additionally, the floors for all model structures are defined as rigid diaphragms. Assuming all structural elements stay in the elastic range and the system is stable during analyses. All model buildings are designed using the structural computational program SAP2000^31^.

### Criteria for regularity in plan

In general, the regularity of the plan can be assessed once the structural model is defined. The criteria for plan regularity are outlined in EN 1998-1 (4.2.3.2)^32,33^:

- The slenderness of the building should not exceed 4 (λ = L_max_/L_min_).
- The structural inherent eccentricity should be less than 30% of the torsional radius (e_nx_≤ 0.30R_x_; e_ny_≤ 0.30R_y_).
- The torsional radius should be greater than the radius of gyration of the floor mass in plan (R_x_ ≥ r; R_y_ ≥ r).

Based on these criteria, the model building used to validate the proposed method is categorized as shown in Table 1. The T-shape, L-shape, C-shape, and H-shape of the model buildings are regular in plan, while wall-shape 1 and wall-shape 2 are irregular due to asymmetrical shear wall placements in the plan. Currently, the proposed model is being tested for design eccentricity validation in both regular and irregular buildings.

**Table 1:** Plan irregularity check for model buildings.

Model building	Structural eccentricity		Frequency ratio		Radius of gyration	Torsional radius		Regularity check	
	|e_nx_|_max_	|e_ny_|_max_	Ω_x_	Ω_y_	r	R_x_	R_y_	0.30R_x_ ≥ e_nx_ 0.30R_y_ ≥ e_ny_	R_x_≥r R_y_≥r
T-shape									
3-story	0.00	0.47	1.08	1.08	14.29	15.47	15.43	✓	✓
7-story	0.00	0.63	1.09	1.08		15.53	15.47	✓	✓
12-story	0.00	0.71	1.09	1.08		15.58	15.49	✓	✓
L-shape									
3-story	0.25	0.25	1.08	1.07	14.29	15.38	15.36	✓	✓
7-story	0.39	0.39	1.08	1.08		15.45	15.42	✓	✓
12-story	0.44	0.44	1.08	1.08		15.37	15.36	✓	✓
C-shape									
3-story	0.33	0.00	1.06	1.09	14.29	15.09	15.50	✓	✓
7-story	0.49	0.00	1.06	1.10		15.12	15.66	✓	✓
12-story	0.68	0.00	1.06	1.11		15.15	15.79	✓	✓
h-shape									
3-story	0.13	0.14	1.06	1.08	14.29	15.07	15.45	✓	✓
7-story	0.22	0.21	1.06	1.09		15.09	15.56	✓	✓
12-story	0.31	0.24	1.06	1.10		15.12	15.66	✓	✓
Wall-shape 1									
3-story	11.42	0.00	1.51	2.90	9.68	14.62	28.10	×	✓
7-story	11.48	0.00	1.34	2.09		12.95	20.19	×	✓
12-story	11.58	0.00	1.23	1.75		11.95	16.93	×	✓
Wall-shape 2									
3-story	4.47	0.00	0.57	0.51	9.68	5.49	4.97	×	×
7-story	4.48	0.00	0.70	0.64		6.75	6.21	×	×
12-story	4.49	0.00	0.76	0.71		7.40	6.85	×	×

## Analysis methods & applied ground motion

**Fig. 7:**
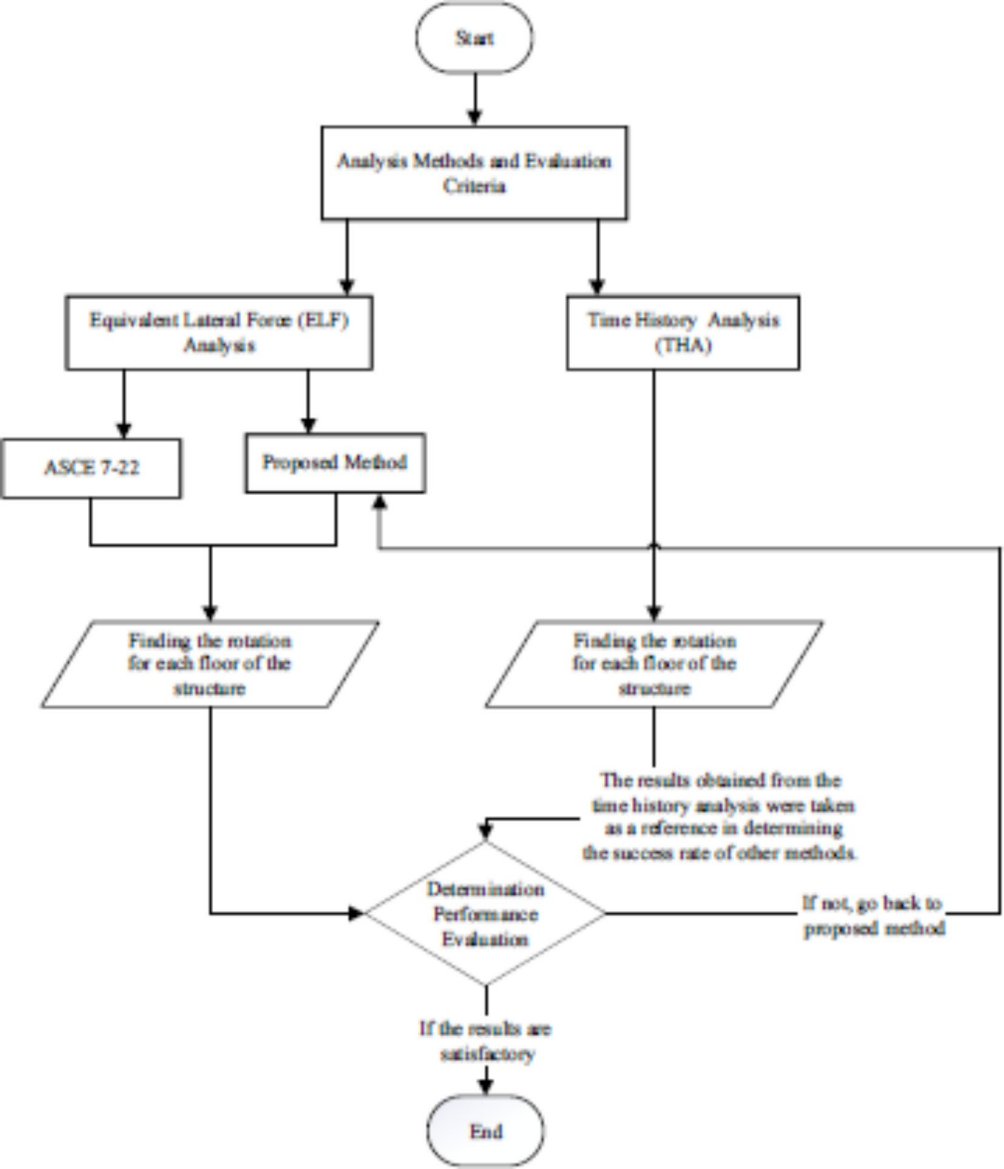
Analysis methods and evaluation criteria.

To account for accidental torsional eccentricity, the same torsional definition is used with ASCE 7–22 which is 5% percent of building dimension perpendicular to the applied earthquake direction. Torsional amplification factor (A_x_) is found and applied if it is higher than 1 and less than 3. If it is higher than 3, the load-bearing system or structural design is encouraged to be modified. All floors are assumed to have rigid diagram effects. Two torsional amplification factors are used for the proposed method, respectively representing (A_x_)_x_ and (A_x_)_y_ for the x- and y- directions, unlike those defined from ASCE 7–22. For the evaluation of the proposed method, analysis methods and evaluation criteria are given in the flow chart, see Fig. 7.

### Equivalent lateral force method

Equivalent Lateral Force (ELF) Method is widely used and a beneficial procedure for approximating the dynamic effects on the structure due to dynamic loadings such as earthquakes or strong winds. The ELF procedure is given in detail in Sect. 12.8 of ASCE 07–22. This procedure’s main goal is to employ static loads on a structure with particular magnitudes and directions until it reaches close approximation dynamic loading effects with dynamic analyses like Spectrum Analysis (SA) or Time History Analysis (THA). At the final stage of this procedure, it is possible to estimate base shear and the horizontal distribution of seismic forces to various structure levels. For the estimation of lateral loads, it is not required to compute the center of stiffness. The obtained equivalent lateral loads can then be applied to each floor level of the structure to acquire the equivalent dynamic effects regardless of performing dynamic analyses.

### Time history analysis

It is a numerical method to estimate the dynamic response of structures while the actual time-varying loads like earthquake excitations are applied. Unlike simplified static analysis assuming dynamic loads remain constant throughout, it instantaneously obtains the dynamic structural response under time-varying force or acceleration. It also offers various advantages compared to other methods: more realistic dynamic behavior/representation, and more accurate evaluation of the safety of a structure. For these advantages, in this study, the dynamic responses of the model buildings are obtained by using bidirectional time history analyses under severe real-life saved earthquake excitations such as Erzincan, Van, Kocaeli, and Kahramanmaras earthquake data. Those results are accepted as references for evaluation and comparison of the proposed design eccentricity and the current code provision.

### Applied ground motion

**Fig. 8:**
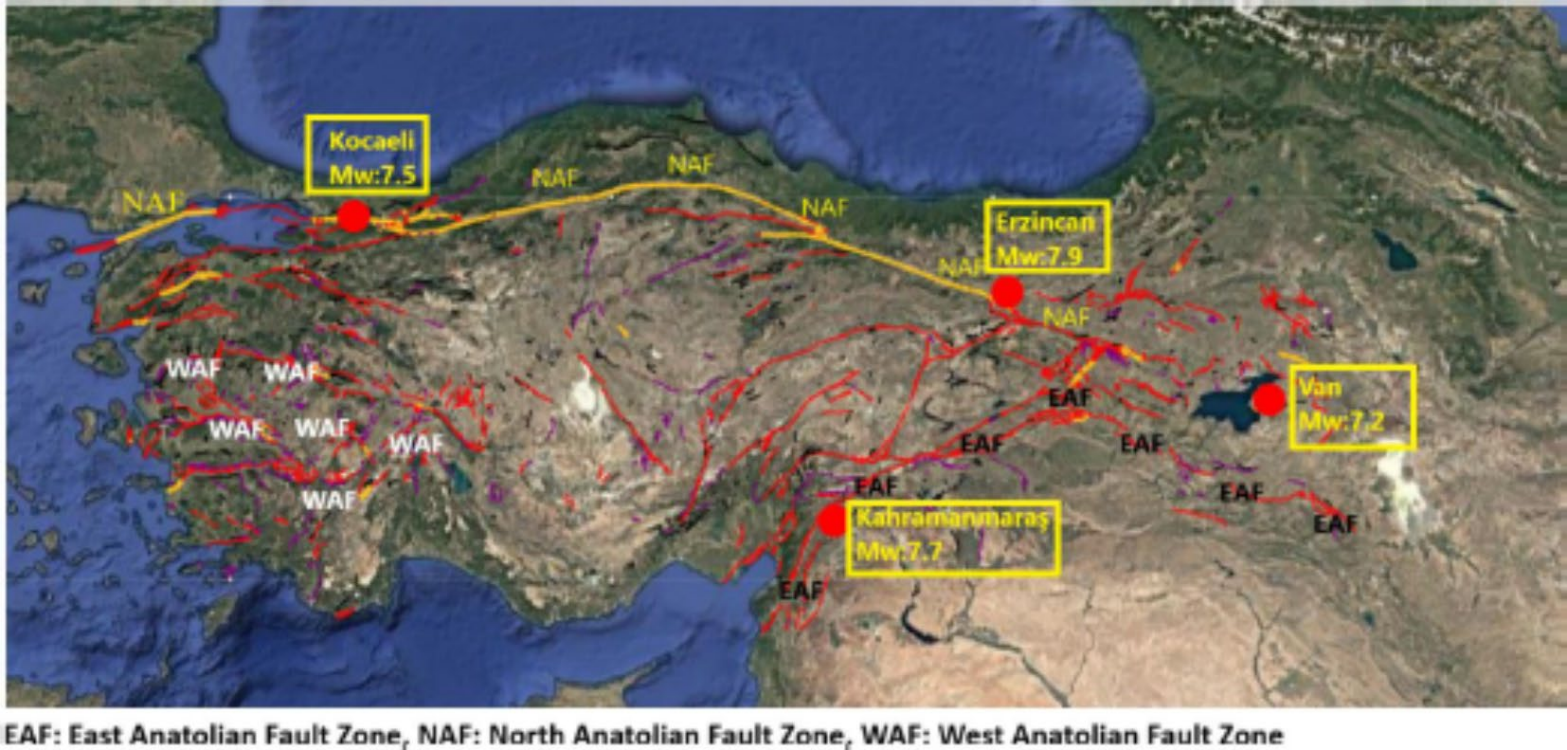
Four historical devastating earthquake records in Türkiye: (**a**) earthquake epicenter and (**b**) active earthquake fault and location. (This figure was generated using active fault locations and earthquake characteristic data obtained from Turkish institutions^34,35^ and is displayed on Google Earth^36^).

Each of the eighteen buildings was analyzed using Equivalent Lateral Force (ELF) and Time History Analysis (THA) methods. All analyses were made in the SAP2000 software^31^. In the THA analysis, the records of the most four severe earthquakes respectively the Erzincan earthquake in 1939; the Kocaeli earthquake in 1999; the Van earthquake in 2011; and the Kahramanmaraş earthquake in 2023 that occurred in Turkey were used, see Fig. 8. The number of earthquake records was limited to four because increasing the number of records and model buildings may make the article difficult to comprehend and follow. The general characteristics of these earthquakes are given in Table 2.

**Table 2:** General characteristics of devastating historical earthquakes in Türkiye.

Province in Türkiye	Recording database	Station No.	Magnitude (M_w_)	Depth (km)	Date	Site Classification according to Eurocode-8
Kahramanmaraş	AFAD^37^	3129	7.7	8.6	2023	B
Van	AFAD^37^	6503	7.2	19.2	2011	C
Kocaeli	AFAD ^37^	8101	7.5	15.9	1999	C
Erzincan	PEER^38^	821	7.9	20	1939	C

**Fig. 9:**
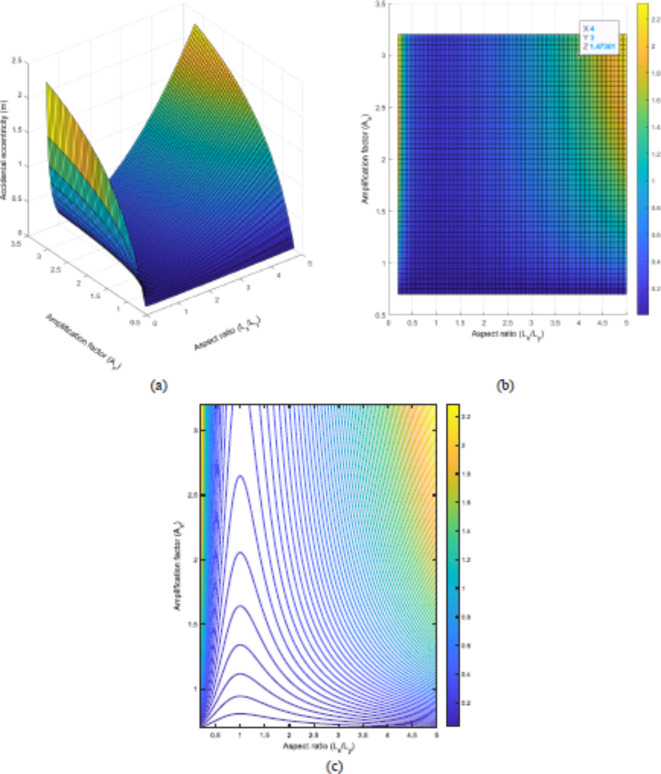
The N-S and E-W Components of the four devastating historical earthquakes that occurred in Türkiye; (**a**) Kahramanmaras, (**b**) Van, (**c**) Kocaeli, and (**d**) Erzincan earthquakes.

The SeismoSignal^39^ program is used to correct the baseline and filter earthquake raw records obtained from the AFAD and PEER databases. The data from SeismoSignal and the code design spectra are then compared and matched using the SeismoMatch^40^ program. Following this process, the earthquake records are utilized in the linear modal time history analysis in the SAP 2000 program. Acceleration vs. time graphs are also provided in Fig. 9.

## Simulation results and discussions

The obtained results have been divided into two main categories: analytical and numerical results. The analytical part involves the evaluation of the proposed eccentricity formula mathematically. The numerical results, on the other hand, consist of an analysis of eighteen buildings with six different floor plans. The dynamic analysis of each building is carried out, and then the obtained numerical results are given here in comparison with the current modern code.

### Analytical results

**Fig. 10:**
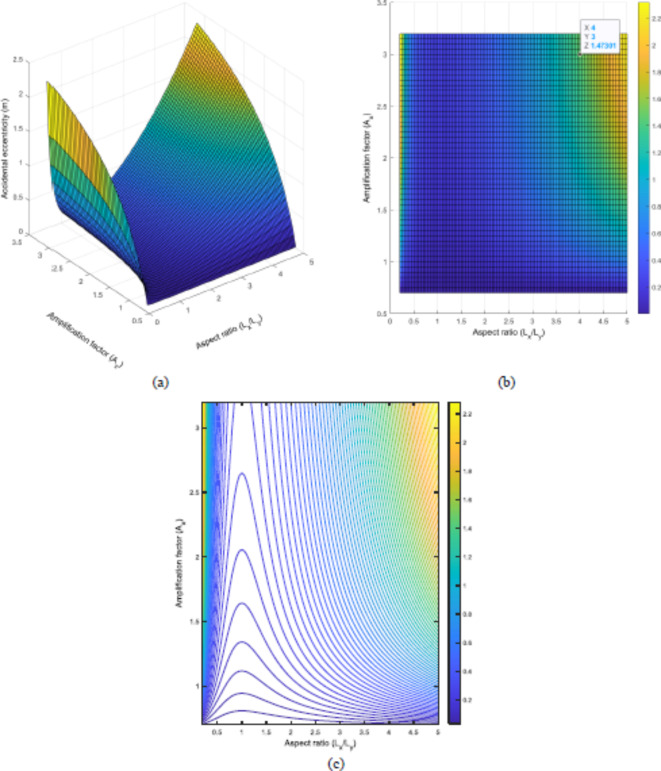
Change in accidental eccentricity by using amplification factor (A_x_) and aspect ratio (L_x_/L_y_) of the model building once $\Omega$ = 1, L_x_ = 1 and L_y_=1; (**a**) 3D graph, (**b**) up-down perspective view of the 3D graph, and (c) contour plot.

To evaluate the proposed method in determining and representing the accidental eccentricity, important parameters such as, frequency ratio, aspect ratio, and torsional amplification factors are used to compute the graphs using MATLAB^41^. Change in the accidental eccentricity by varying amplification factor (A_x_) and building aspect ratio (L_x_/L_y_) is illustrated in Fig. 10. A_x_ is bounded from 0.7 to 3 that it can get as maximum values defined as the ASCE 7–22 torsional definition and the aspect ratio gets values from 0.2 to 5 that stay in the realistic limitation while Eurocode-8^33^ suggest that it should be got 4 as the maximum value.

In a study by De-la-Colina et al. in 2016^42^, it was stated that the aspect ratio of a building’s plan plays a crucial role in determining accidental eccentricity. Buildings with square plans (aspect ratio of 1) are less susceptible to accidental eccentricity than those with rectangular plans (aspect ratio higher or lower than 1). This observation is in line with other studies^9,43,44^. Additionally, buildings with square plans have a higher amplification factor (A_x_) than those with rectangular plans, which can cause higher accidental eccentricity. However, the maximum accidental eccentricity occurs in rectangular buildings, as seen in Fig. 10a and c. In Fig. 10b, the maximum accidental eccentricity obtained by the proposed method at A_s_ = 4 is selected, with a value of 1.47 m. This value is crucial in determining the maximum frequency ratio ($\Omega$) that the building can safely withstand.

The frequency ratio ($\Omega$) is another crucial parameter that helps determine how accidental eccentricity is affected by it. According to De la Llera and Chopra^45^ in 1995, the frequency ratio ($\Omega$) plays a vital role in determining accidental eccentricity. They classified the system based on $\Omega$ as follows: If $\:\Omega\le\:1$, it is a torsionally flexible system (TFS), if $\:1<\Omega\le\:2.5$, it is a torsionally medium system (TMS), which is neither flexible nor stiff., and if $\:\Omega>2.5$, the system is a torsionally stiff system (TSS). When it is $\:0.75\le\:\le\:1.25$, it becomes vital in terms of determining design eccentricity. The same classifications are used in the present study.

**Fig. 11:**
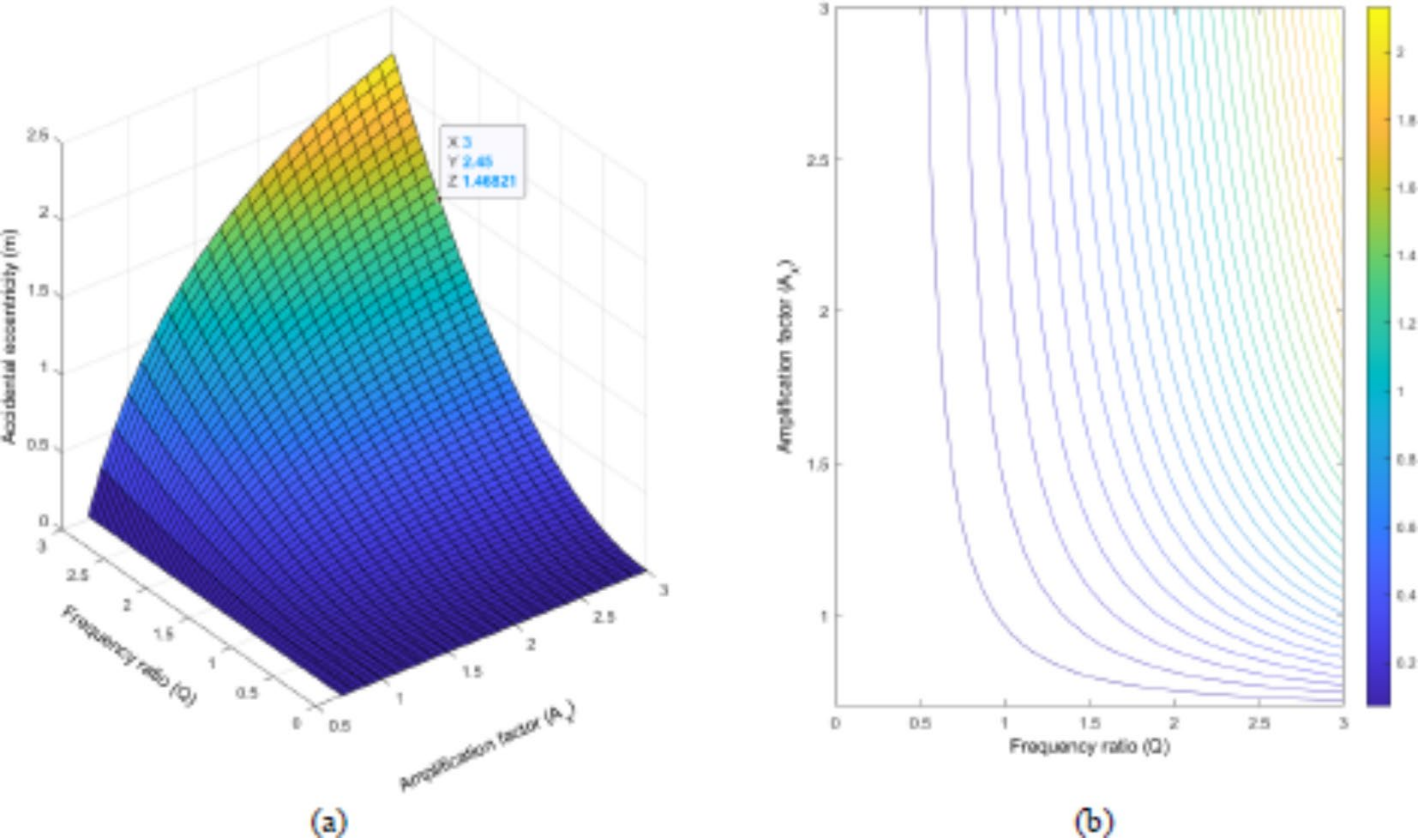
Change in accidental eccentricity by using Amplification factor (A_x_) and Frequency ratio ($\Omega$) of the model building once A_s_=1, L_x_=1 and L_y_=1; (**a**) 3D graph and (**b**) contour plot.

As depicted in Fig. 11a, the observed accidental eccentricity is lower if the system is TFS, whereas TSS leads to a significantly higher accidental eccentricity. Hence, the selected value of 1.47 m determines the boundary limit for the frequency ratio. If the value of $\Omega$ is 2.45 when A_x_=3, then the observed accidental eccentricity is almost equivalent to that value. In addition, looking into Fig. 11b, when the system is TFS ($\:\Omega\le\:1$), the Amplification factor (A_x_) increases rapidly. Once $\Omega$ is greater than 2.5, A_x_ becomes stable. These observations align with the Eurocode 8 and other researches done before.

**Fig. 12:**
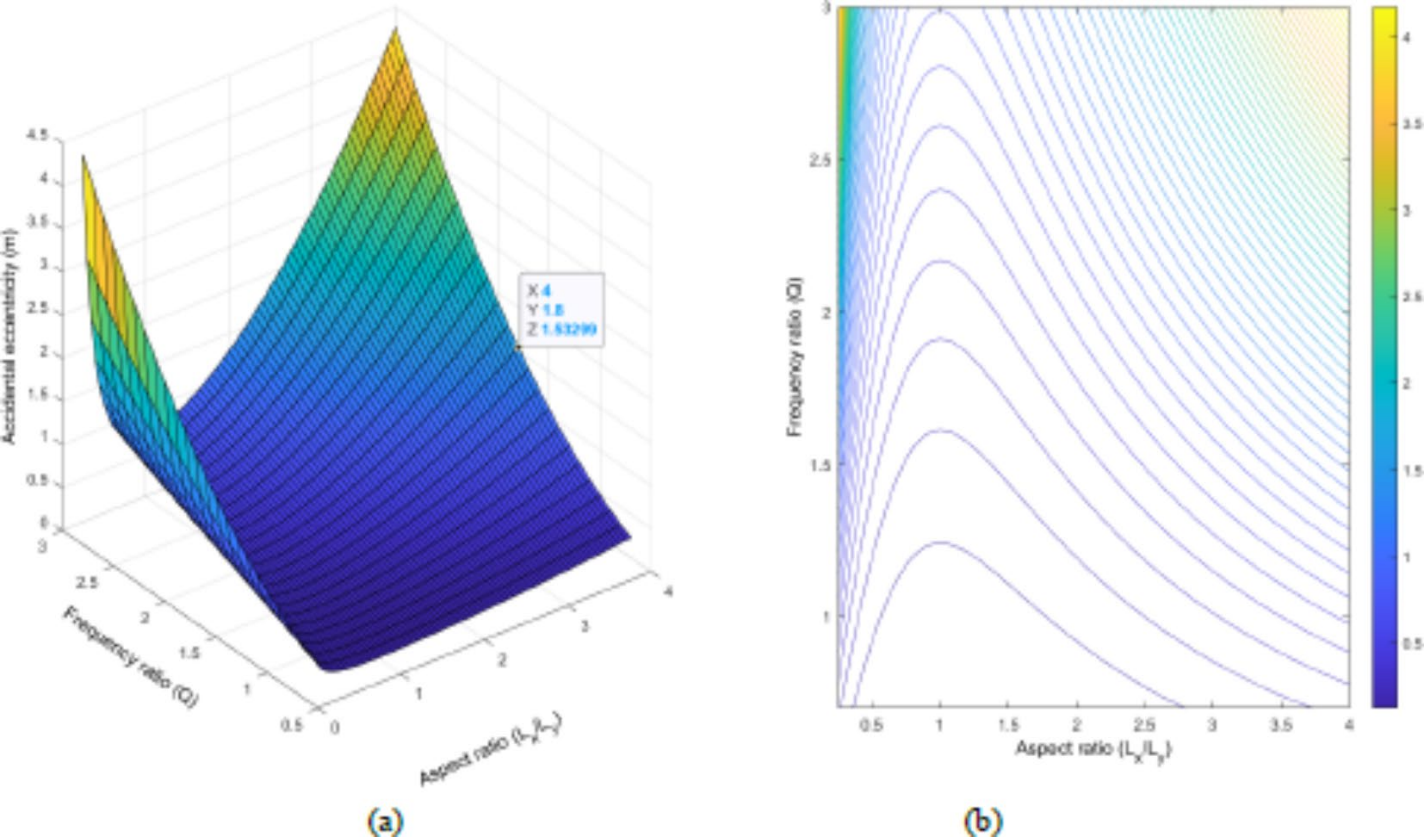
Change in accidental eccentricity by using Frequency ratio ($\Omega$) and Aspect ratio (L_x_/L_y_) of the model building once A_s_ is varying, but L_x_=1 and L_y_=1; (**a**) 3D graph and (**b**) contour plot.

When A_s_ is varying as seen in Fig. 12a, the selected value of 1.47 m determines the frequency ratio’s boundary limit, which is now $\Omega$=1.8. This finding is similar to that of study done by (Mohamed and Mehana in 2020). According to the proposed methodology, the system should have a frequency ratio not exceeding 1.8. Furthermore, a similar observation is made that the more the building has higher aspect ratios, the more it will undergo accidental eccentricity. When it comes to Fig. 12b, shows that the building with a square shape in plan (A_s_=1) has a higher frequency ratio that can give approximately the same accidental eccentricity as the rectangular ones having a lower frequency ratio. This circumstance leads us to conclude that the square buildings show better performance in terms of accidental eccentricity as compared to their relevant counterparts.

### Numerical results

To analyze the performance of the proposed method, we have selected eighteen buildings with six different floor plans that have either geometric eccentricity or are inherently eccentric due to un-uniformly distributed load-bearing member placements. These buildings are subjected to time history analyses (THAs) under bidirectional loading cases by four historically devastating earthquakes that have taken place in Turkey. The period of the model buildings is provided in Table 3.

**Table 3:** Model building period (sn).

Model building	Number of stories	Period		
		x-dir.	y-dir.	θ-dir.
T-Shape	3-story	0.406	0.405	0.375
	7-story	1.091	1.086	1.003
	12-story	1.957	1.946	1.795
L-Shape	3-story	0.409	0.408	0.380
	7-story	1.095	1.093	1.013
	12-story	1.964	1.957	1.809
C-Shape	3-story	0.4	0.411	0.378
	7-story	1.067	1.106	1.009
	12-story	1.942	2.024	1.832
h-Shape	3-story	0.396	0.405	0.375
	7-story	1.093	1.06	1.004
	12-story	1.934	2.004	1.828
Wall-Shape 1	3-story	0.245	0.221	0.432
	7-story	0.82	0.754	1.176
	12-story	1.664	1.543	2.179
Wall-Shape 2	3-story	0.243	0.466	0.161
	7-story	0.806	1.256	0.602
	12-story	1.626	2.304	1.318

**Fig. 13:**
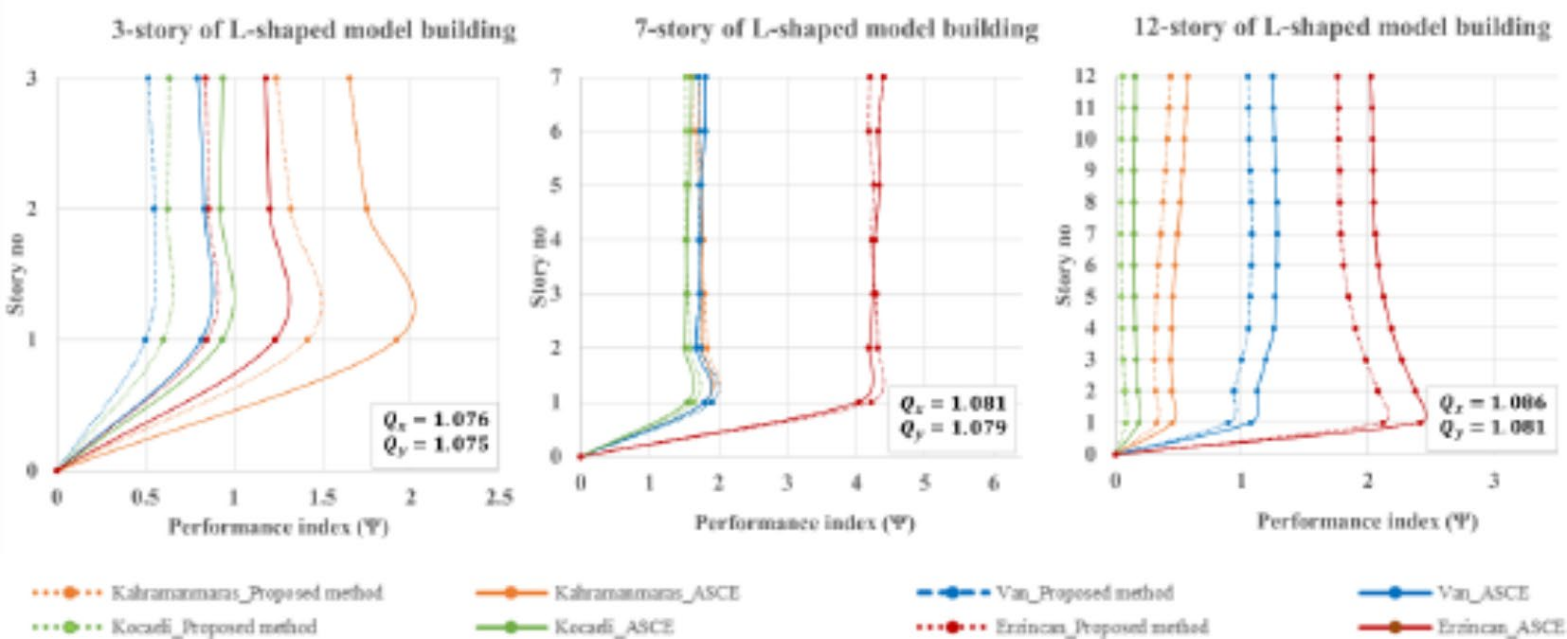
Performance index for L-shaped buildings during four historically devastating earthquakes.

The proposed method and ASCE method are used to determine the design eccentricity by using the ELF procedure. Additional torsional moments are calculated for both methods and applied to the center of mass (CM) of the building model. Torsional responses (rotation) are obtained at the CM and compared to the results obtained through THAs, which are considered as references. For evaluation purpose, performance indexes are obtained through Eq. (34).

34 $$\:{\psi\:}_{Proposed}=\frac{{Rot}_{Proposed}-{Rot}_{THA}}{{Rot}_{THA}};\:or\:{\psi\:}_{ASCE}=\frac{{Rot}_{ASCE}-{Rot}_{THA}}{{Rot}_{THA}}\:\:\:\:\:$$

where $\:{Rot}_{Proposed}$ and $\:{Rot}_{ASCE}$; $\:{\psi\:}_{Proposed}$ and $\:{\psi\:}_{ASCE}$ represent the obtained rotational responses and performance indexes by using respectively the proposed method and ASCE 7–22 provision and $\:{Rot}_{THA}$ denotes for the rotational response by obtaining through time history analysis (THA). Performance indexes for L-shaped buildings, including three-, seven-, and twelve-story buildings, are illustrated in Fig. 13.

It is essential to note that the structural and ground motion complexities may lead to unpredictable damages and responses in a structure. In this study, only structural complexities (one- or two-way eccentric) are considered. Therefore, we set performance criteria by aiming to achieve more secure responses as close to the THAs responses as possible by excluding the other reasons that can lead to accidental eccentricity.

In the case of L-shaped buildings, the frequency ratios for both orthogonal directions are slightly higher than one, indicating a torsionally medium system (TMS). Accurately estimating the torsional response of L-shaped buildings can be challenging. However, the proposed method has demonstrated better performance in predicting torsional response under various earthquake loading scenarios compared to the conventional ASCE method. Specifically, for a 3-story L-shaped building in the Kahramanmaras 2023 earthquake, the maximum performance index (Ψ) is approximately 1.5 for the proposed method, whereas the ASCE method yields around 2, see Fig. 13. Similar trends are observed for the 12-story model building, with the proposed method consistently outperforming the ASCE method under all earthquake circumstances. Notably, for a 7-story building, the ASCE method shows comparable or slightly better performance on the lower floors, while the proposed method exhibits better performance on the upper floors.

**Fig. 14:**
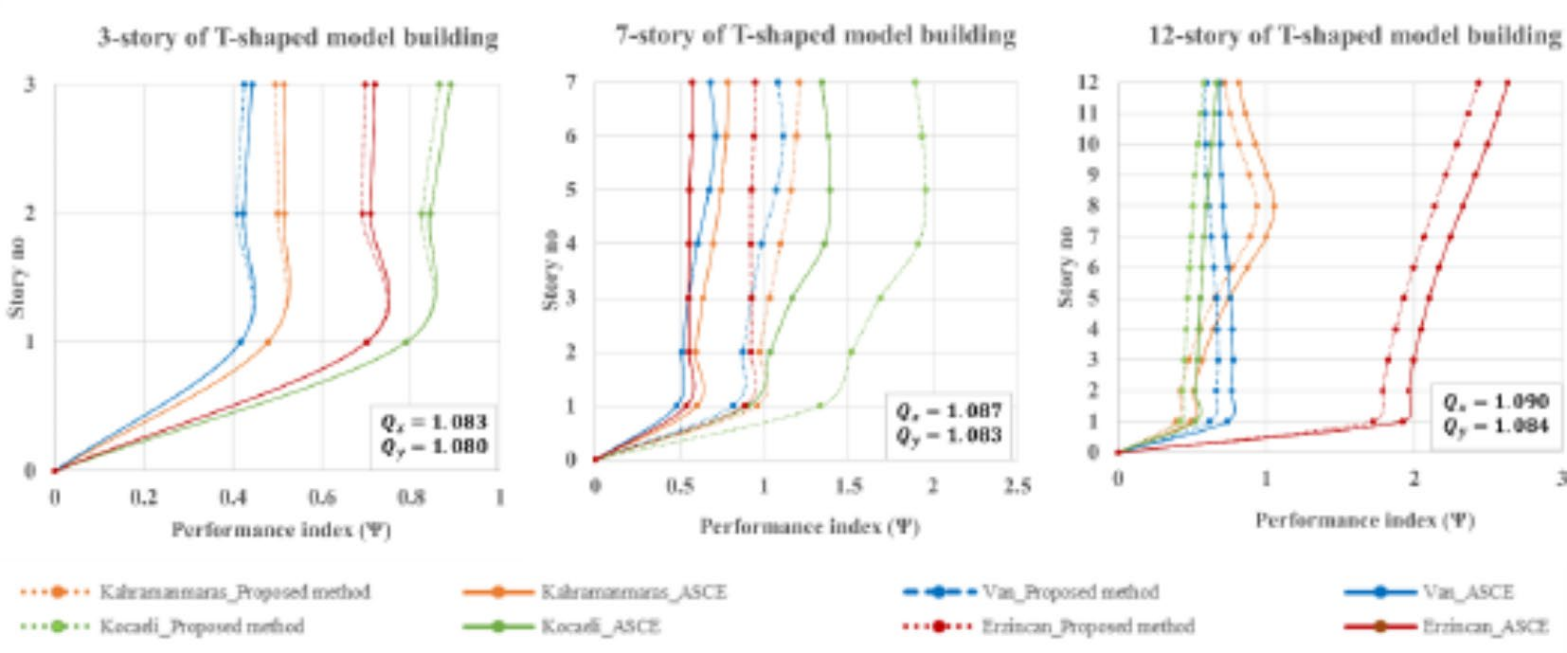
Performance index for T-shaped buildings during four historically devastating earthquakes.

The performance of T-shaped buildings is comparable to that of L-shaped buildings. According to Fig. 14, the proposed method outperforms low-rise (3-story) and high-rise (12-story) buildings. However, for mid-rise (7-story) buildings, the ASCE method is more effective for all earthquake scenarios. In the case of L-shaped buildings, it can be concluded that mid-rise structures are more critical in terms of design eccentricity.

**Fig. 15:**
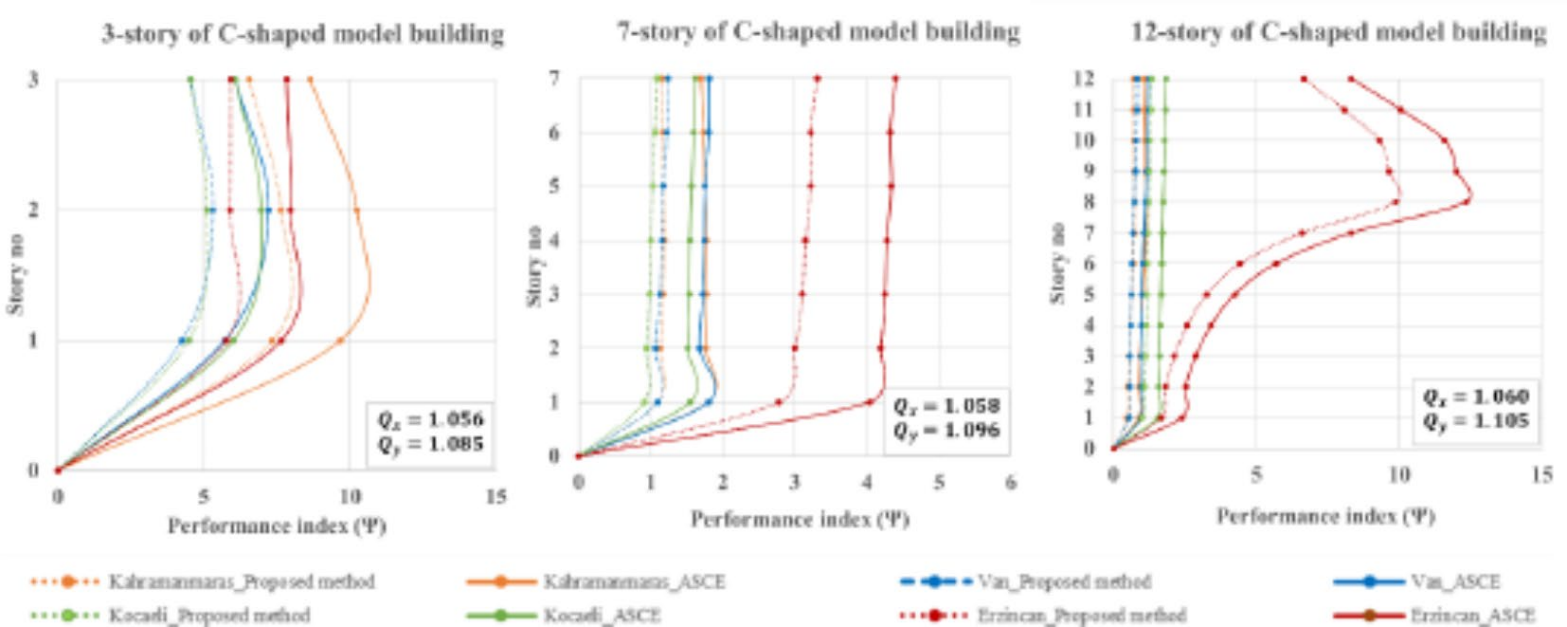
Performance index for C-shaped buildings during four historically devastating earthquakes.

When it comes to C-shaped buildings, for all types of (low-, mid-, and high-rise) buildings, the proposed method shows better performance as compared to the ASCE method, see Fig. 15. It is interesting to note that the maximum phi values range from 10 to 15 for 3-story and 12-story C-shaped buildings, in contrast, the 7-story building follows a similar trend with lower phi values reaching a maximum of 4.2. The performance indexes for 7- and 12-story occur under bidirectional Erzincan earthquake loading. For 3-story buildings, the Kahramanmaras earthquake emerges as critical. This situation highlights the role of earthquake dynamic characteristics in the performance of existing or proposed methods.

**Fig. 16:**
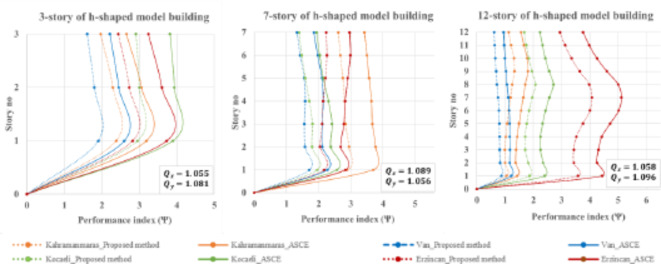
Performance index for h-shaped buildings during four historically devastating earthquakes.

In addition, Fig. 16 illustrates the performance indexes of h-shaped buildings. The frequency ratios range between 1 and 1.1, indicating that all buildings are still within the threshold of being TMS. The Erzincan and Kahramanmaras earthquakes respectively play significant roles in the torsional response contribution for 7-, 12-story, and 3-story buildings. Overall, the proposed method outperforms the ASCE method for low-, mid-, and high-rise h-shaped buildings.

Wall-shaped 1 buildings have irregular plans according to Eurocode 8, as shown in Table 1. As a result, the conventional design approach based on the building code may not be suitable for this type of structure. These buildings typically have frequency ratios around 0.5, indicating that they are torsionally flexible systems (TFS). This flexibility makes them more sensitive to accidental eccentricities. This study evaluates unaltered wall-shape 1 buildings to assess the effectiveness of the proposed system.

**Fig. 17:**
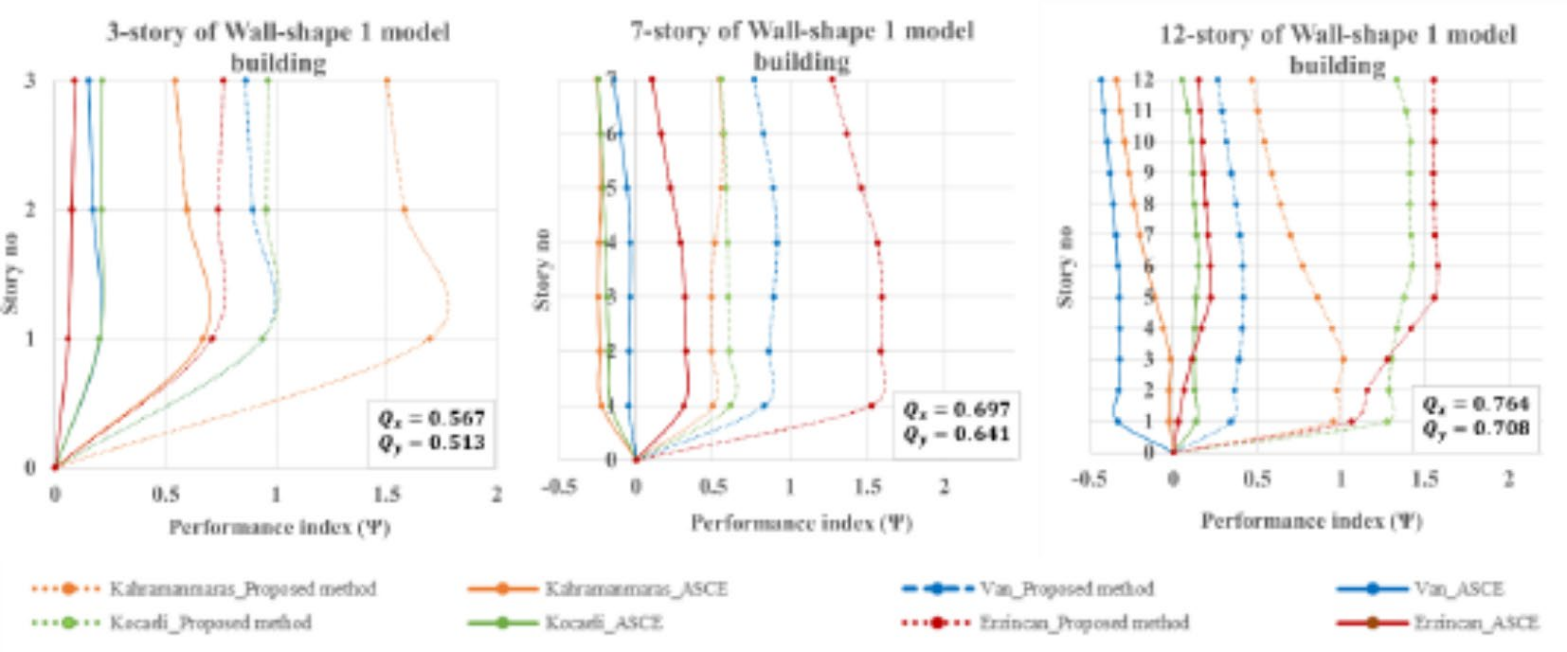
Performance index for Wall-shape 1 buildings during four historically devastating earthquakes.

As observed in Fig. 17, it can be inferred that the ASCE method is unsatisfactory in predicting torsional response for Wall-shaped 1 seven- and twelve-story buildings during the Kahramanmaras and Van earthquakes. Furthermore, the ASCE method also fails to predict the torsional response for the seven-story building during the Erzincan earthquake, indicating its vulnerability to accidental eccentricity prediction. The placement of non-symmetrical shear walls in the plan sufficiently increases the eccentricity, making the ASCE method an insecure method for predicting torsional irregularity effects. On the contrary, the proposed method provides torsional responses higher than the THAs of the four historical earthquakes in predicting torsional response. The maximum Ψ value (roughly 1.7) occurs in the three-story building during the Kahramanmaras earthquake, and subsequently, the rest of the torsional responses are close to the THAs but not less than that. This demonstrates that the proposed method is an acceptable and validated approach for predicting torsional irregularity effects.

**Fig. 18:**
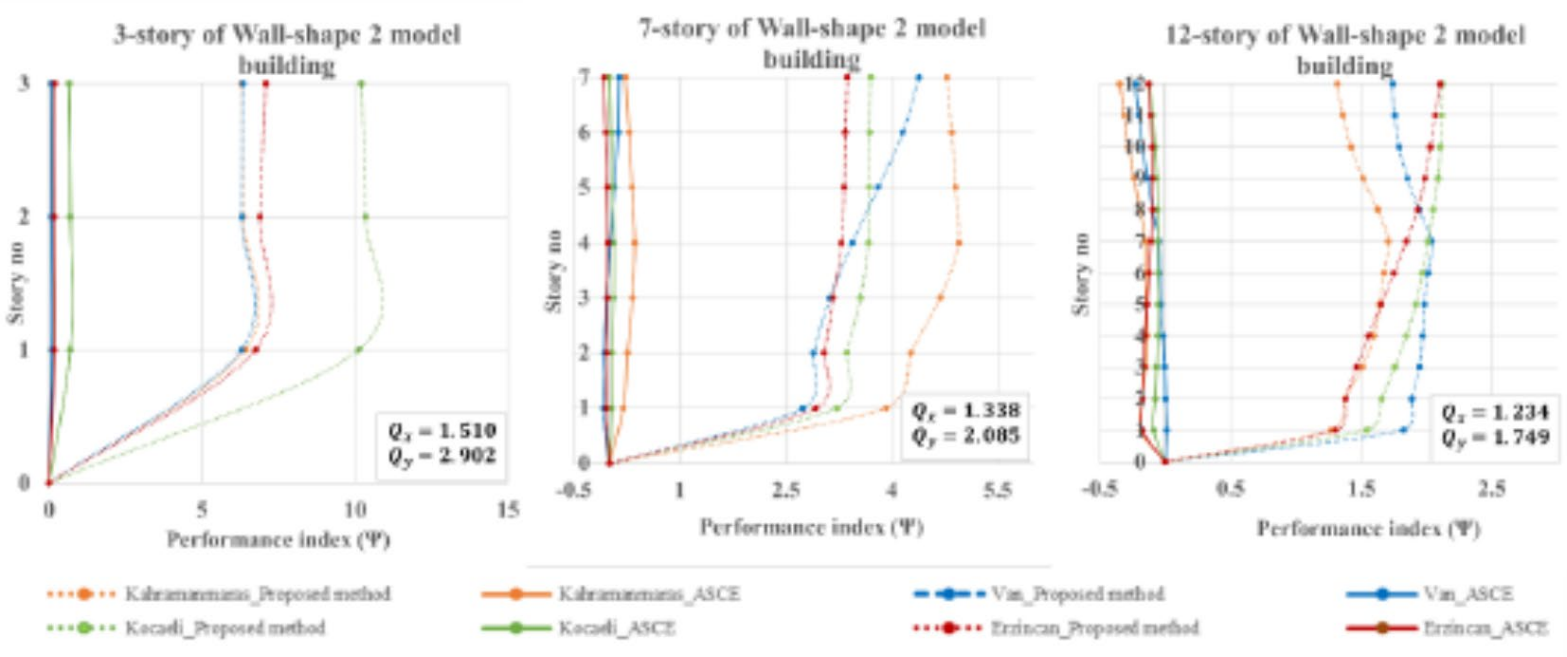
Performance index for Wall-shape 2 buildings during four historically devastating earthquakes.

Similar to Wall-shape 1 buildings, Wall-shaped 2 buildings also have irregular plans according to Eurocode 8, see Table 1. As a result, the conventional design approach based on the building code may not be suitable for this type of structure. These buildings typically have frequency ratios between 1.234 and 1.510 for the x-direction and between 1.749 and 2.902 for the y-direction indicating that they can be defined as either torsionally medium system (TMS) or torsionally stiff systems (TFS). The TFS buildings have resilience against torsional irregularity. However, In Fig. 18, additional accidental eccentricity obtained by the ASCE method is deemed unreliable and insufficient in accounting for torsional eccentricity in the Wall-shape 2 building that is eccentric due to unsymmetric shear wall placements in the plan. The ASCE method is successful in predicting accidental eccentricity for low-rise buildings under all earthquake loading cases, however appears to fall short in most cases, except the Erzincan earthquake, where the accidental torsional eccentricity assumptions are less than required for mid-rise and high-rise buildings. The difference reaches approximately 35% less as compared to the results obtained by THAs. On the other hand, the proposed method outperforms code-based design eccentricity for all earthquake cases, demonstrating its success in predicting the occurrence of torsional eccentricity.

## Conclusion

The study provides an alternative formula for torsional eccentricity that incorporates the frequency ratio and effective radius of gyration. While a number of studies have shown that the frequency ratio (uncoupled torsional frequency/translation frequency ($\:{\Omega}_{x}$ or $\:{\Omega}_{y}$) is a significant factor in estimating the accidental eccentricity effects of a structure^14,27,44–46^, no methodology has yet been proposed that includes it. The proposed method not only predicts accidental torsional effects but also provides insight into the structural torsional behavior, such as flexibility or stiffness, which is not possible with the current code torsional definition. Additionally, the formula can be easily manipulated and interpreted with the information of slenderness or torsional radius of a structure, as required by the current earthquake codes like Eurocode 8. Furthermore, the proposed formula eliminates the need to calculate geometric eccentricity to compute the design eccentricity when bidirectional earthquake loadings are taken into account, which is more realistic than unidirectional loading.

The proposed method for the design eccentricity has undergone an extended validation evaluation, with analysis results divided into two parts: analytical and numerical. The analytical results examine the proposed method’s evaluation in determining and representing the accidental eccentricity, using important parameters such as frequency ratio, aspect ratio, and torsional amplification factors. In the numerical results, linear analyses were conducted on eighteen buildings with six different floor plans, representing low, medium-height, and high-rise structures. The analyses utilized the linear THA method with four devastating historical earthquakes that occurred in Turkish history, namely Kahramanmaras (2023), Van (2011), Kocaeli (1999), and Erzincan (1939). The obtained results were compared to code provisions. Based on the numerical results, it appears that the ASCE method may not be sufficient for computing design eccentricity, particularly in Wall-shape 1 and Wall-shape 2 model buildings that are eccentric due to asymmetric shear wall placements.

It is important to note that the proposal presented is currently provisional. The authors aim to offer an alternative method for determining design eccentricity but do not intend to modify the definition of torsional irregularity. In addition, we, the authors, plan to examine the performance of the proposed method under nonlinear time history analyses and more complex building plans or designs for future research.

## Electronic supplementary material

Below is the link to the electronic supplementary material.

Supplementary Material 1

## Data Availability

The datasets generated during and/or analysed during the current study are available from the corresponding author on reasonable request.

## List of symbols

AFAD: Disaster and emergency management presidency of Turkiye
A_s_: Aspect ratio
ASCE: American Sociaty of Civil Engineering
A_x_: Torsional irregularity coefficient
(A_x_)_x_and (A_x_)_y_: Torsional amplification factors for the x- and y-directions
CM: Centr of mass
CR: Center of rigidity
CRot: Center of rotation
e_acx_: Accidental eccentricity in the x direction
e_acy_: Accidental eccentricity in the y direction
(e_d_): Design eccentricity under bidirectional loading case (x and y-directions)
e_d1_ and e_d2_: Code-based design eccentricities for earthquake loading through in the x-direction
e_d1i_ and e_d2i_: Code-based design eccentricities for earthquake loading through in the x-direction at the i-th floor
(e_d_)_x_: Design eccentricity under unidirectional loading case (only x-direction)
(e_d_)_y_: Design eccentricity under unidirectional loading case (only y-direction)
ELF: Equivalent lateral force.
e_n_: Absolute geometric eccentricity: $\:{e}_{n}=\sqrt{{{e}_{nx}}^{2}+{{e}_{ny}}^{2}}$
e_nx_: Geometric eccentricity in the x direction
e_ny_: Geometric eccentricity in the y direction
Eurocode: Europe earthquake code
$\:{F}_{e}$: Equivalent lateral earthquake shear force
F_e1i_ and F_e2i_: Code-based equivalent earthquake force at the i-th floor
$\:{F}_{s}$: Equivalent strain force
$F_{s}^{\prime }$: Equivalent strain force after applied force
J_0_: Polar mass moment of inertia
$$\:{k}_{\theta\:}$$: Torsional stiffness
L_x_ and L_y_: Building dimension in the relevent direction
M, C, and K: Mass, damping and stiffness matrices
M_d1i_ and M_d2i_: Resultant torsional moment due to acciental eccentricity i-th floor
$\:{M}_{\theta\:}$: Torque (torsional moment) with respect to the CM
PEER: Pacific earthquake engineering research center
r: Radius of gyration
r_ef_: Effective radius of gyration
r_x_ and r_y_: Radius of gyration components in the relevent directions
$\:{Rot}_{ASCE}$: Obtained rotational responses by ASCE method
$\:{Rot}_{Proposed}$: Obtained rotational responses by proposed method
$\:{Rot}_{THA}$: Obtained rotational response by obtaining through time history analysis (THA)
TBEC: Turksih Building Earthquake Code
THA: Time history analysis
TIBs: Torsionally irregular buildings
W: Modified input vector
$\:x\left(t\right)$: Building response displacement in the x-direction without consideration of eccentrictiy
$\:{x\left(t\right)}_{max}$ and $\:{\theta\:\left(t\right)}_{max}$: Peak response of the time history analysis in the x- and $\theta$-direction
$\:\varDelta\:x\left(t\right)$: Additional displacement response due to eccentricity
$\:{\ddot{x}}_{g}\left(t\right)$: Earthquake input vector
$\Omega$: Coupled frequency ratio
$\Omega _{x}$ and $\Omega _{y}$: Coupled frequency ratio (fundamental torsional frequency/translation frequency for related direction)
$\:(\delta\:\left(t\right),\:\dot{\delta\:}\left(t\right),\:\ddot{\delta\:}\left(t\right))$: Displacement, velocity and acceleration vectors
$\:{w}_{x},\:{w}_{y}\:and\:{w}_{\theta\:}$: Frequency (Hz) x-; y-lateral directions and $\:\theta\:$-torsional direction respectively
$\:{\delta\:}_{avg}$: Average displacement at the building edge that gives the maximum change in response at level i + 1 floor
$\:{\delta\:}_{min}\:and\:{\delta\:}_{max}\:$: Respectively the minimum and maximum displacements
$\:{\psi\:}_{ASCE}$: Performance index for the ASCE method
$\:{\psi\:}_{Proposed}$: Performance index for the proposed method
$\:\gamma\:$: Proposed torsional irregularity coefficient (PTIC)
