# Supplementary material for: An alternative recommendation for design eccentricity by consideration of uncoupled frequency ratio
Source: Sci Rep. 2024 Dec 28;14:30673. doi: 10.1038/s41598-024-75465-3 (PMC11680927; doi:10.1038/s41598-024-75465-3)
Supplement: Supplementary file 1 — Supplementary Material 1 [file 41598_2024_75465_MOESM1_ESM.docx]

**Appendix A:** Derivation of the torsional stiffness


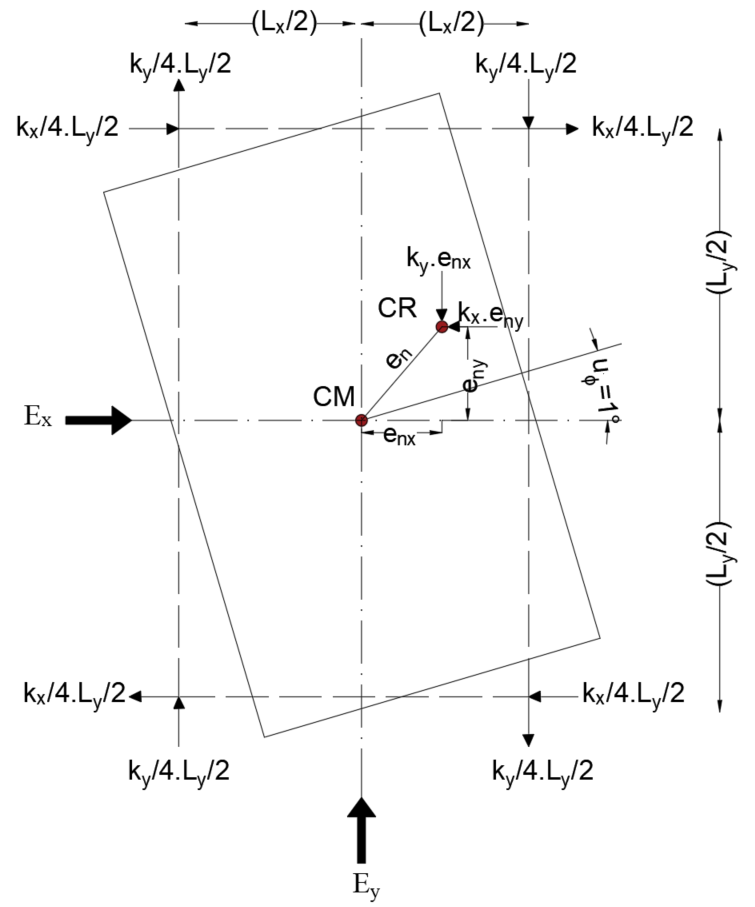


**Figure A1**. Torsional stiffness calculation for a two-way eccentric one-story building.

Where E_x_ and E_y_ represent equivalent earthquake loadings applied to the center of mass (CM); k_x_ and k_y_ stand for total equivalent stiffness of the ith floor for x- and y-directions; and e_nx_ and e_ny_ are the geometric eccentricity for the relevant directions.

$$k_{x}=\sum_{1}^{i=n} k_{xi} and k_{y}=\sum_{1}^{i=n} k_{yi} n is the number of columns in a related story.$$

For a simple building plan with four equal square columns, $k_{x1}=k_{x2}=k_{x3}=k_{x3}=\frac{k_{x}}{4}$ and $k_{y1}=k_{y2}=k_{y3}=k_{y3}=\frac{k_{y}}{4}$.

In order to compute torsional stiffness (k_θ_), unit rotation uϕ=1 is applied to the CM. Then torsional stiffness (k_θ_) becomes by equilibrating resisting forces as:

$$k_{\theta}=4.\left( \frac{k_{x}}{4}.\frac{L_{y}}{2}.\frac{L_{y}}{2} \right)+4.\left( \frac{k_{y}}{4}.\frac{L_{x}}{2}.\frac{L_{x}}{2} \right)+k_{x}e_{ny}.e_{ny}+k_{y}e_{nx}.e_{nx}$$

Rearranging the Equation, it becomes as

$$k_{\theta}=k_{x}.\frac{{L_{y}}^{2}}{4}+k_{y}.\frac{{L_{x}}^{2}}{4}+k_{x}e_{ny}^{2}+k_{y}e_{nx}^{2}$$

**Appendix B:** Determining of the center of rigidity

All model buildings are designed and analyzed in the structural software program (Sap2000). For the determination of the structural eccentricities of i^th^ level, the equations provided below can be used^33,47^

|  | $\left( e_{nx} \right)_{i}=-\frac{\left( R_{z} \right)_{i}\left( \left( F_{y} \right)_{i}=1 \right)}{\left( R_{z} \right)_{i}\left( \left( M \right)_{i}=1 \right)}; \left( e_{ny} \right)_{i}=\frac{\left( R_{z} \right)_{i}\left( \left( F_{x} \right)_{i}=1 \right)}{\left( R_{z} \right)_{i}\left( \left( M \right)_{i}=1 \right)}$ |  |
| --- | --- | --- |

where $\left( R_{z} \right)_{i}\left( \left( F_{x} \right)_{i}=1 \right)$ and $\left( R_{z} \right)_{i}\left( \left( F_{y} \right)_{i}=1 \right)$ are respectively the rotation of the i^th^ storey about vertical axes due to static load $\left( F_{x} \right)_{i}=1$ and $\left( F_{y} \right)_{i}=1$ in the x and y directions and $\left( R_{z} \right)_{i}\left( \left( M \right)_{i}=1 \right)$ is the rotation due to torsional moment about the vertical axis. The forces $\left( F_{x} \right)_{i}$ and $\left( F_{y} \right)_{i}$ and the moment $\left( M \right)_{i}$ are applied in the center of mass in the i^th^ story.

**Appendix C:** Detailed calculation information for T-shape and Wall-shape 1 buildings

**Table C1.** Detailed properties belong to the T-shape buildings: 3-, 7- and 12-story.

| Model | | Story | Building  dimension (m) | | Geometric eccentricity | | | Frequency (Hz) | | | Frequency ratio | | Effective radius of gyration | | | | Torsional amplification factor | |
| --- | --- | --- | --- | --- | --- | --- | --- | --- | --- | --- | --- | --- | --- | --- | --- | --- | --- | --- |
|  |  | no | x- dir. | y- dir. | e_nx_ (m) | e_ny_ (m) | e_n_ (m) | x- dir. | y- dir. | Ɵ- dir. | Ω_x_ | Ω_y_ | r_x_ | r_y_ | r | r_ef_ | (A_x_)_x_ | (A_x_)_y_ |
| T-shape building | 3-story | 1 | 35 | 35 | 0.000 | 0.160 | 0.160 | 2.463 | 2.471 | 2.668 | 1.083 | 1.080 | 10.104 | 10.104 | 14.289 | 14.449 | 0.952 | 0.884 |
|  |  | 2 |  |  | 0.000 | 0.333 | 0.333 |  |  |  |  |  |  |  |  | 14.622 | 0.933 | 0.893 |
|  |  | 3 |  |  | 0.000 | 0.470 | 0.470 |  |  |  |  |  |  |  |  | 14.758 | 0.980 | 0.885 |
|  | 7-story | 1 |  |  | 0.000 | 0.146 | 0.146 | 0.917 | 0.921 | 0.997 | 1.087 | 1.083 |  |  |  | 14.434 | 1.156 | 0.857 |
|  |  | 2 |  |  | 0.000 | 0.292 | 0.292 |  |  |  |  |  |  |  |  | 14.581 | 1.140 | 0.857 |
|  |  | 3 |  |  | 0.000 | 0.392 | 0.392 |  |  |  |  |  |  |  |  | 14.681 | 1.143 | 0.868 |
|  |  | 4 |  |  | 0.000 | 0.465 | 0.465 |  |  |  |  |  |  |  |  | 14.754 | 1.137 | 0.871 |
|  |  | 5 |  |  | 0.000 | 0.523 | 0.523 |  |  |  |  |  |  |  |  | 14.812 | 1.146 | 0.880 |
|  |  | 6 |  |  | 0.000 | 0.575 | 0.575 |  |  |  |  |  |  |  |  | 14.863 | 1.152 | 0.882 |
|  |  | 7 |  |  | 0.000 | 0.627 | 0.627 |  |  |  |  |  |  |  |  | 14.916 | 1.143 | 0.915 |
|  | 12-story | 1 |  |  | 0.000 | 0.146 | 0.146 | 0.511 | 0.514 | 0.557 | 1.090 | 1.084 |  |  |  | 14.434 | 1.223 | 1.225 |
|  |  | 2 |  |  | 0.000 | 0.291 | 0.291 |  |  |  |  |  |  |  |  | 14.580 | 0.964 | 0.727 |
|  |  | 3 |  |  | 0.000 | 0.391 | 0.391 |  |  |  |  |  |  |  |  | 14.680 | 0.965 | 0.738 |
|  |  | 4 |  |  | 0.000 | 0.463 | 0.463 |  |  |  |  |  |  |  |  | 14.751 | 0.967 | 0.782 |
|  |  | 5 |  |  | 0.000 | 0.516 | 0.516 |  |  |  |  |  |  |  |  | 14.805 | 0.974 | 0.843 |
|  |  | 6 |  |  | 0.000 | 0.556 | 0.556 |  |  |  |  |  |  |  |  | 14.845 | 0.982 | 0.923 |
|  |  | 7 |  |  | 0.000 | 0.588 | 0.588 |  |  |  |  |  |  |  |  | 14.877 | 1.216 | 1.225 |
|  |  | 8 |  |  | 0.000 | 0.614 | 0.614 |  |  |  |  |  |  |  |  | 14.903 | 0.978 | 0.727 |
|  |  | 9 |  |  | 0.000 | 0.637 | 0.637 |  |  |  |  |  |  |  |  | 14.926 | 0.980 | 0.738 |
|  |  | 10 |  |  | 0.000 | 0.660 | 0.660 |  |  |  |  |  |  |  |  | 14.948 | 0.988 | 0.782 |
|  |  | 11 |  |  | 0.000 | 0.683 | 0.683 |  |  |  |  |  |  |  |  | 14.972 | 1.008 | 0.843 |
|  |  | 12 |  |  | 0.000 | 0.711 | 0.711 |  |  |  |  |  |  |  |  | 15.000 | 1.010 | 0.923 |

**Table C2.** Detailed properties belong to the Wall-shape 1 buildings: 3-, 7- and 12-story.

| Model | | Story | Building  dimension (m) | | Geometric eccentricity | | | Frequency (Hz) | | | Frequency ratio | | Effective radius of gyration | | | | Torsional amplification factor | |
| --- | --- | --- | --- | --- | --- | --- | --- | --- | --- | --- | --- | --- | --- | --- | --- | --- | --- | --- |
|  |  | no | x- dir. | y- dir. | e_nx_ (m) | e_ny_ (m) | e_n_ (m) | x- dir. | y- dir. | Ɵ- dir. | Ωx | Ωy | r_x_ | r_y_ | r | r_ef_ | (A_x_)_x_ | (A_x_)_y_ |
| Wall-shape 1 building | 3-story | 1 | 30 | 15 | 4.470 | 0.000 | 4.470 | 4.088 | 4.519 | 2.316 | 0.567 | 0.513 | 8.660 | 4.330 | 9.682 | 14.152 | 0.931 | 3.917 |
|  |  | 2 |  |  | 4.450 | 0.000 | 4.450 |  |  |  |  |  |  |  |  | 14.133 | 0.907 | 3.725 |
|  |  | 3 |  |  | 4.308 | 0.000 | 4.308 |  |  |  |  |  |  |  |  | 13.991 | 0.848 | 3.261 |
|  | 7-story | 1 |  |  | 4.482 | 0.000 | 4.482 | 1.220 | 1.327 | 0.850 | 0.697 | 0.641 |  |  |  | 14.164 | 0.907 | 3.469 |
|  |  | 2 |  |  | 4.494 | 0.000 | 4.494 |  |  |  |  |  |  |  |  | 14.177 | 0.849 | 3.010 |
|  |  | 3 |  |  | 4.327 | 0.000 | 4.327 |  |  |  |  |  |  |  |  | 14.010 | 0.836 | 2.660 |
|  |  | 4 |  |  | 4.116 | 0.000 | 4.116 |  |  |  |  |  |  |  |  | 13.798 | 0.819 | 2.346 |
|  |  | 5 |  |  | 3.876 | 0.000 | 3.876 |  |  |  |  |  |  |  |  | 13.559 | 0.815 | 2.083 |
|  |  | 6 |  |  | 3.621 | 0.000 | 3.621 |  |  |  |  |  |  |  |  | 13.303 | 0.776 | 1.778 |
|  |  | 7 |  |  | 3.392 | 0.000 | 3.392 |  |  |  |  |  |  |  |  | 13.075 | 0.758 | 1.486 |
|  | 12-story | 1 |  |  | 4.488 | 0.000 | 4.488 | 0.601 | 0.648 | 0.459 | 0.764 | 0.708 |  |  |  | 14.170 | 0.827 | 3.127 |
|  |  | 2 |  |  | 4.510 | 0.000 | 4.510 |  |  |  |  |  |  |  |  | 14.193 | 0.827 | 2.778 |
|  |  | 3 |  |  | 4.367 | 0.000 | 4.367 |  |  |  |  |  |  |  |  | 14.050 | 0.816 | 2.473 |
|  |  | 4 |  |  | 4.195 | 0.000 | 4.195 |  |  |  |  |  |  |  |  | 13.877 | 0.797 | 2.228 |
|  |  | 5 |  |  | 4.017 | 0.000 | 4.017 |  |  |  |  |  |  |  |  | 13.699 | 0.798 | 2.033 |
|  |  | 6 |  |  | 3.842 | 0.000 | 3.842 |  |  |  |  |  |  |  |  | 13.524 | 0.785 | 1.881 |
|  |  | 7 |  |  | 3.671 | 0.000 | 3.671 |  |  |  |  |  |  |  |  | 13.353 | 0.785 | 1.758 |
|  |  | 8 |  |  | 3.504 | 0.000 | 3.504 |  |  |  |  |  |  |  |  | 13.187 | 0.777 | 1.613 |
|  |  | 9 |  |  | 3.338 | 0.000 | 3.338 |  |  |  |  |  |  |  |  | 13.020 | 0.770 | 1.482 |
|  |  | 10 |  |  | 3.164 | 0.000 | 3.164 |  |  |  |  |  |  |  |  | 12.847 | 0.764 | 1.324 |
|  |  | 11 |  |  | 2.983 | 0.000 | 2.983 |  |  |  |  |  |  |  |  | 12.665 | 0.744 | 1.148 |
|  |  | 12 |  |  | 2.819 | 0.000 | 2.819 |  |  |  |  |  |  |  |  | 12.501 | 0.748 | 1.000 |
